# Epigenetic precision diagnostics of traditional Chinese medicine (TCM) syndrome differentiation: a pilot study of atrial fibrillation with qi-yin deficiency syndrome based on 5-hydroxymethylcytosine signatures in extracellular vesicle DNA from plasma

**DOI:** 10.1186/s13020-025-01267-y

**Published:** 2026-05-27

**Authors:** Shaowei Fan, Haoyu Chen, Hangyu Chen, Bai Du, Baixin Zhen, Xianglong Chen, Lei Zhang, Xiaxuan Li, Maimaitiyasen Duolikun, Long Chen, Han Gao, Shuqing Shi, Xiaohan Zhang, Yangang Wang, Yuanhui Hu, Jian Lin

**Affiliations:** 1 Department of Cardiology, Guang’anmen Hospital, China Academy of Chinese Medical Sciences, Beijing, 100053 China; 2 School of Graduate, Hebei University of Chinese Medicine, Xinshi South Road No. 326, Qiaoxi District, Shijiazhuang, 050091 Hebei China; 3 Department of Pharmacy, Peking University Third Hospital, Beijing, 100191 China; 4 College of Pharmacy, Xinjiang Medical University, Urumqi, 830011 China; 5 School of Information and Intelligent Engineering, University of Sanya, Sanya, 572022 Hainan China; 6 School of Information and Communication Engineering, Hainan University, Haikou, 570228 Hainan China; 7 Key Laboratory of Tropical Biological Resources of Ministry of Education, School of Pharmaceutical Sciences, Hainan University, Haikou, 570100 China; 8 Synthetic and Functional Biomolecules Center, Beijing National Laboratory for Molecular Sciences, Peking University, Beijing, 100871 China; 9 Peking University Third Hospital Cancer Center, Beijing, 100191 China; 10 Department of Internal Medicine, Guang’anmen Hospital, China Academy of Chinese Medical Sciences, Beijing, 100053 China; 11 Beijing University of Chinese Medicine Third Affiliated Hospital, Anwai Xiaoguan Street No.51, Chaoyang District, Beijing, 100029 China

**Keywords:** Atrial fibrillation, Traditional Chinese medicine (TCM) syndrome differentiation, Qi-Yin deficiency syndrome, 5-hydroxymethylcytosine, Machine learning

## Abstract

**Background:**

Syndrome differentiation in Traditional Chinese Medicine (TCM) is pivotal to clinical practice and dictates the efficacy of medicinal treatments. However, precision diagnostic models for TCM syndromes, constructed from biomarkers such as metabolites and proteins, have failed to achieve high precision. Recent studies have highlighted a strong link between TCM and epigenetics, an area that remains largely unexplored in TCM diagnosis. Taking atrial fibrillation (AF) with Qi-Yin deficiency syndrome (QYDS) as an example, we utilized a type of epigenetic sequencing technology called 5hmC-Seal and integrated it with various machine learning models to develop an Epigenetic Differential Syndrome (Epi-DS) technology for identifying epigenetic biomarkers. This approach is crucial for developing more accurate diagnostic models for traditional Chinese medicine syndromes and for advancing the modernization of traditional Chinese medicine.

**Methods:**

In this study, we conducted a single-center, prospective study involving two independent cohorts (cohort 1 and cohort 2) in AF, including QYDS and non-Qi-Yin deficiency syndrome (NQYDS). Next, we utilized 5hmC-Seal to obtain the patients’ 5hmC genome-wide profiles in plasma extracellular vesicles DNAs (evDNAs). Meanwhile, a variety of sophisticated machine learning algorithms were employed across three datasets—training, validation, and external cohorts (the training and validation sets constituting cohort 1 and the external cohort constituting cohort 2) to construct and validate QYDS diagnosis model.

**Results:**

Based on the hydroxymethylation profile of the QYDS in AF, we have successfully constructed a disease-phenotype-molecule biological network for AF. At the molecular level, we identified nine characteristic 5hmC markers for the QYDS in AF and successfully established a diagnostic model for this syndrome. In Cohort 1’s training set, the area under the receiver operating characteristic curve (AUC) was as high as 0.984, with a sensitivity of 0.976 and a specificity of 1.000. In validation set, the AUC was 0.949, with a sensitivity of 0.952 and a specificity of 0.952. In the independent external validation cohort 2, the AUC was as high as 0.934, with a sensitivity of 0.886 and a specificity of 0.919. Moreover, the diagnostic model we built based on symptoms and molecular markers achieved an AUC value of 0.864 in an independent external cohort.

**Conclusions:**

A novel precision diagnostic approach of TCM Syndrome Differentiation was established based on Epi-DS. The disease-phenotype-molecule network we have constructed reveals the epigenetic foundation of TCM and has identified molecular diagnostic markers for the QYDS in AF. This provides an example for understanding the molecular basis of TCM syndrome differentiation and for integrated traditional Chinese and Western medicine diagnosis.

**Supplementary Information:**

The online version contains supplementary material available at 10.1186/s13020-025-01267-y.

## Introduction

Traditional Chinese Medicine (TCM) syndrome differentiation is the foundation for formulating TCM prescriptions, ensuring personalized treatment [1, 2]. However, since syndrome typing is determined based on relatively subjective information such as symptoms and signs collected by physicians, it often leads to unclear diagnosis and misdirected treatment, ultimately resulting in suboptimal therapeutic outcomes. Therefore, leveraging modern technology to clarify TCM syndrome differentiation is of paramount importance. With the continuous advancement of the modernization of TCM, numerous studies have established extensive databases of modern objective data, including genomics and metabolomics, relevant to TCM syndrome typing. Despite numerous attempts to utilize this information to construct diagnostic models for TCM syndrome typing, a robust and precise model has yet to be achieved [3, 4]. Therefore, the modernization of TCM urgently requires linking the relatively subjective process of syndrome differentiation with objective and precise data, such as molecular omics. This not only enables the creation of multi-level biological networks that encompass diseases, phenotypes, and molecules but also facilitates the discovery of molecular markers for precise diagnosis of TCM syndrome typing, thereby achieving the modernization of TCM syndrome differentiation.

The rise of epigenetics has opened new possibilities for the modernization of TCM [5–7]. Epigenetics, via mechanisms such as DNA methylation and demethylation, sustains a subtle dynamic equilibrium in gene expression without sequence alteration, profoundly impacting expression trends [8]. Although the epigenetic regulation of gene expression resonates with the holistic outlook and dynamic yin–yang balance of TCM, the two frameworks are by no means identical [9]. Mounting evidence indicates that environmental and lifestyle factors—long recognized in TCM as key triggers for syndrome differentiation—can also reshape the epigenome, thereby influencing disease initiation and progression [5]. Recent studies have further demonstrated that a growing number of Chinese herbal medicines exert their therapeutic effects by modulating the epigenetic landscape, underscoring the intimate connection between traditional Chinese medicine and epigenetics [10–12]. Furthermore, TCM’s syndrome differentiation encapsulates the pathological equilibrium during disease, often foreshadowing its trajectory [13]. Epigenetics reflects the body’s response to external changes, regulating gene transcription to influence disease progression, echoing TCM’s diagnostic typing. The regulatory role provides a molecular explanation for the syndrome differentiation in TCM and enhances our understanding of its biological basis. Consequently, the epigenetic differential syndrome (Epi-DS) constructed based on the epigenetic molecular landscape provided by epigenetic sequencing may offer a new perspective for the modernization of traditional Chinese medicine.

5-hydroxymethylcytosine (5hmC) is a key intermediate in the DNA demethylation process and plays a crucial role in numerous physiological and pathological processes, often referred to as the “sixth base” of DNA [14, 15]. It exhibits a unique genomic distribution and exerts distinct functional influences, setting it apart from 5-methylcytosine (5mC) in its contribution to gene regulation [16, 17]. Research conducted by our group and others has demonstrated that the 5hmC profiles of cell-free DNA (cfDNA) in plasma can serve as epigenetic biomarkers for a range of human diseases, such as coronary heart disease, myocardial injury, esophageal cancer, lung cancer, lymphoma, neurodegenerative diseases, and more [18–23]. In recent advancements, research has revealed that extracellular vesicles (EVs) are pivotal in the transmission of bioactive molecules over both short and long distances, thereby facilitating robust intercellular communication [24, 25]. These EVs possess the remarkable capacity to traverse the endothelial barriers and integrate into the bloodstream, offering a stable and informative snapshot of the body’s disease status [26, 27]. Notably, the presence of EVs in plasma has been recognized as a treasure trove for the diagnostic and prognostic evaluation of cardiovascular conditions, heralding a new era in the early detection and management of heart-related diseases [28, 29]. Furthermore, research has indicated that EVs contain genetic materials such as genomic DNA and mitochondrial DNA. Through the transfer of DNA, they play a pivotal role in maintaining cellular homeostasis [30, 31]. Therefore, the detection of extracellular vesicle DNAs (evDNAs) in the blood can facilitate the diagnosis, drug response monitoring, and molecular analysis of diseases [32, 33].

Qi-Yin deficiency syndrome (QYDS) is the most common TCM syndrome type among patients with AF. Clinical practice has shown that many traditional Chinese patent medicines exhibit significant therapeutic effects in treating AF patients with QYDS [34–37]. Therefore, this study takes the QYDS in AF as an entry point and applies an advanced sequencing technology—5hmC-Seal [18], which is a sensitive method based on chemical labeling that allows for comprehensive 5hmC-Seal analysis of evDNAs in plasma. We conducted a study on the genome-wide 5hmC levels of 196 AF patients, including 99 with QYDS patients and 97 with non-Qi-Yin deficiency syndrome (NQYDS) patients. The purpose of our research is to explore the 5hmC distribution characteristics in AF with QYDS, thereby constructing a disease-phenotype-molecular biological network, and identifying new biomarkers for the diagnosis of AF with QYDS. Our research has constructed an epigenetic molecular map for TCM syndromes, providing a new perspective for the objective study of TCM syndromes. Additionally, we constructed diagnostic models by combining TCM symptoms with modern molecular markers, providing a new direction for modern integrated traditional Chinese and Western medicine diagnosis. Epi-DS, as a novel precision diagnostic approach for TCM syndromes, offers a fresh perspective on the modernization and internationalization of TCM.

## Materials and methods

### Design of study

**Fig. 1:**
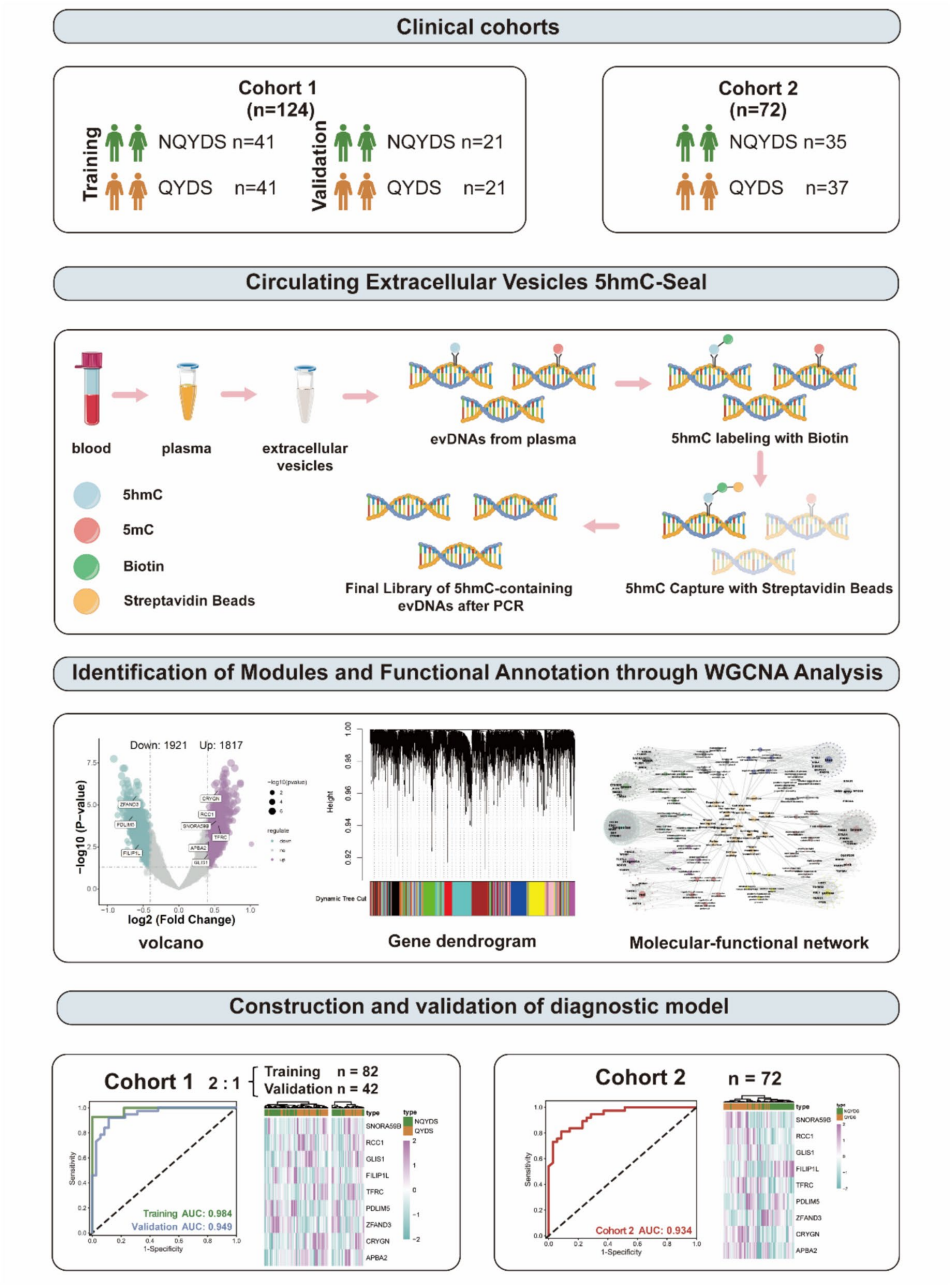
Schematic overview of the study. This study enrolled a total of 196 AF patients. The study population was divided into two cohorts: Cohort 1 (n = 124) was further divided into the training and the validation in a 2:1 ratio for analysis. 5hmC expression profiles were obtained using the 5hmC-Seal technique. WGCNA was utilized to perform modular functional enrichment analysis of differentially expressed genes. Subsequently, machine learning techniques were applied to the expression profiles of the training to develop a diagnostic model, which was then validated using the expression profiles of the validation. Additionally, the diagnostic model was further validated in a double-blind manner using the genome-wide 5hmC expression profiles from Cohort 2

This study recruited patients with AF who had either QYDS or NQYDS from Guang’anmen Hospital of China Academy of Chinese Medical Sciences in two independent cohorts (Cohort 1 and Cohort 2). The cohort 1 was divided into the training and the validation in a 2:1 ratio. Participants were recruited from July 2023 to December 2023 for 5hmC feature analysis and to construct a disease-phenotype-molecular network to elucidate the molecular functions of QYDS. Finally, biomarkers were identified, and a diagnostic model was constructed based on this. Cohort 2 served as an independent external test set, with participants recruited from January 2024 to April 2024, to validate the accuracy of the diagnostic model. The study has been approved by the Ethics Committee of Guang’anmen Hospital, China Academy of Chinese Medical Sciences (No. 2023-132-KY). The overall workflow and details of each step of this study are shown in Fig. 1.

### Participants

The diagnosis of AF is based on the “2020 ESC Guidelines for the diagnosis and management of AF developed in collaboration with the European Association for Cardio-Thoracic Surgery (EACTS)” [38]. The diagnostic criteria for AF include the presence of an electrocardiographic event with the disappearance of P waves and an absolutely irregular RR interval (without atrioventricular block), documented by a standard 12-lead ECG or a single-lead ECG for more than 30 s, or by a Holter ECG recording. The diagnosis of QYDS is based on the QYDS scale (supplementary material S1), which was established by a panel of 10 senior TCM experts using the Delphi expert consultation method. This scale includes 10 core items, and the scoring is completed through negotiation between the physician and the patient. A total score greater than 8 points is considered a diagnosis of QYDS. Tongue appearance and pulse diagnosis are used as auxiliary diagnostic tools. Those who do not meet the aforementioned diagnostic criteria for QYDS are all classified as NQYDS. Moreover, given the subjective nature of symptoms, all TCM practitioners involved in this study received unified training. In cases where the diagnosis was controversial or unclear, multiple physicians would be organized to discuss the matter. In addition, we also conducted random sampling reviews of the diagnostic results of some patients to ensure the accuracy and consistency of the diagnosis.

Inclusion criteria are as follows: (1) aged between 35 and 79 years, gender not specified; (2) conforming to the diagnostic criteria for AF; (3) meeting the diagnostic criteria for QYDS or NQYDS; (4) the patient is informed, volunteers to participate in this study, and has signed an informed consent form. Exclusion criteria are as follows; (1) Patients with rheumatic heart disease, congenital heart disease, hypertrophic cardiomyopathy, restrictive cardiomyopathy, dilated cardiomyopathy, and valvular AF; (2) Patients with acute coronary syndrome, acute cerebral infarction, systolic blood pressure ≥ 180 mmHg and/or diastolic blood pressure ≥ 110 mmHg, and those with severe cardiopulmonary insufficiency; (3) Patients with severe hepatic and renal dysfunction (creatinine clearance < 30 ml/min or active nephritis, serum transaminases ≥ 3 times the upper limit of clinical reference values), hematological disorders, autoimmune diseases, endocrine system diseases, or other serious primary diseases that affect life expectancy; (4) Patients with malignant tumors, acute infections, or other major diseases; (5) Pregnant women, women who have had a miscarriage, or women who are breastfeeding; (6) Patients who cannot cooperate or refuse to cooperate due to illness or other reasons in completing the collection of relevant information.

### Collection of clinical information

Our group has collected basic information, medical history, symptoms, signs, and related indicators of the participants, such as age, height, weight, body mass index (BMI), diastolic blood pressure (DBP), systolic blood pressure (SBP), heart rate (HR), and comprehensive evaluation of cardiac function status, as well as whether they suffer from hyperhomocysteinemia, hyperuricemia, type 2 diabetes, hyperlipidemia, chronic obstructive pulmonary disease (COPD), obstructive sleep apnea, cerebral infarction, and old myocardial infarction. Additionally, we assisted patients in conducting CHA2DS2-VASc score assessments [39] and HASBLED score assessments [40]. We collected these variables specifically to evaluate the baseline match between QYDS and NQYDS and thereby minimize the risk of confounding.

### Sample size calculation

This study conducted a literature review to assess the performance of existing diagnostic models in establishing a molecular diagnostic model for TCM syndromes, revealing that the AUC generally ranges from 0.8 to 0.95 [4, 41]. Therefore, leveraging our research team’s previous experience in constructing diagnostic models for the cardiovascular system [18, 23], we selected an AUC of 0.9 as the benchmark for estimating the required sample size. We calculated the sample size using the formula provided by the Power Analysis and Sample Size (PASS, 2021 edition) software. The ratio of the positive to negative groups was set to 1:1, and the alternative hypothesis AUC was set to 0.9. Additionally, we set 0.75 as a hypothesized value to serve as the standard for testing.

The formula and the calculation process are as follows:

$$n = \frac{{\left[ {Z_{1 - \alpha } \sqrt {p_{0} \left( {1 - p_{0} } \right)} + Z_{1 - \beta } \sqrt {p_{1} \left( {1 - p_{1} } \right)} } \right]^{2} }}{{\left( {p_{1} - p_{0} } \right)^{2} }}$$

Firstly, we establish the values for α andβ at 0.05 and 0.2, respectively. For alpha, the Z score corresponding to a 95% confidence level, Z_1−α_, is approximately 1.96. For β, assuming a power of 80%, Z_1−β_ is roughly 0.84.

Next, we calculate the variance for the baseline proportion *p*0 = 0.75 and the new proportion *p*1 = 0.9:

$$Z_{1 - \alpha } \sqrt {p_{0} \left( {1 - p_{0} } \right)} = 1.96 \times \sqrt {0.75\left( {1 - 0.75} \right)} \approx 0.849$$

$$Z_{1 - \beta } \sqrt {p_{1} \left( {1 - p_{1} } \right)} = 0.84 \times \sqrt {0.9\left( {1 - 0.9} \right)} = 0.252$$

We then sum these products and divide by the difference between *p*1 and *p*0:

$$n = \left( {\frac{0.849 + 0.252}{{0.9 - 0.75}}} \right)^{2} = 53.8756 \approx 54$$

Ultimately, our calculations based on the formula yield a required sample size of 54 × 2 = 108 people. Taking this into account, we endeavored to collect more than this threshold number of participants during the study period. Ultimately, we enrolled a total of 196 patients. Cohort 1 comprised 124 subjects who were randomly split into a training set and a validation set in a 2:1 ratio using R. Cohort 2 comprised 72 subjects. This approach ensured that our sample size was sufficiently large to conduct robust statistical analyses and detect the effect sizes of interest, thereby enhancing the reliability and validity of our research findings.

### Plasma separation and evDNAs extraction

Following our previous experimental methods [42], whole blood samples were obtained through routine venipuncture and collected into cell-free DNA collection tubes (Roche). The tubes were maintained at a temperature of 15 °C to 25 °C. Within 24 h, the plasma was separated by centrifuging the whole blood at 4 °C at 1350 × g for 15 min and then at 4 °C at 13,500 × g for 5 min, and then stored at − 80 °C for future use. Extracellular EVs extraction is performed according to the Exosome Extraction Kit (H-Wayen), involving incubation with reagents and centrifugation at 10,000 × g and 3000 × g for extraction. The extraction and purification of evDNAs are carried out according to the Quick-DNA Extraction Kit (ZYMO). The process involves the addition of BioFluid&Cell Buffer, Proteinase K, Genomic Binding Buffer, g-DNA Wash Buffer, along with incubation and centrifugation at 12,000 × g for extraction and purification. The extracted evDNAs should be stored at − 20 °C. Before the preparation of the 5hmC library, verify the fragment size of all evDNAs samples using nucleic acid electrophoresis.

### Construction of 5hmC library and high-throughput sequencing

In this study, all samples’ 5hmC libraries were constructed using the efficient hmC-Seal technique, as adopted from previous experiments [16]. Following the kit’s protocol, the extracted evDNAs from plasma is subjected to end repair and 3′-adenylation using the KAPA Hyper Prep Kit (KAPA Biosystems), followed by ligation with Illumina-compatible adapters. The ligated evDNAs is then glycosylated by incubation in a solution containing HEPES buffer (pH 8.0), MgCl2, UDP-6-N3-Glc, and β-glucosyltransferase (NEB). Subsequently, DBCO-PEG4-biotin (Click Chemistry Tools) is added and incubated again. The DNA is then purified using the DNA Clean & Concentrator Kit (ZYMO). The purified DNA is incubated with streptavidin beads (Life Technologies) in a buffer containing Tris pH 7.5, EDTA, NaCl, and Tween 20, followed by washing. All binding and washing steps are carried out at room temperature with gentle rotation. The beads are then resuspended in RNase-free water and subjected to 14–16 cycles of PCR amplification. The PCR products are purified using AMPure XP beads (Beckman) according to the manufacturer’s instructions. Finally, the library concentration is measured using the Qubit 3.0 fluorometer (Life Technologies). Libraries that meet the quantification standards are then subjected to paired-end 150 bp high-throughput sequencing on the NovaSeq 6000 platform.

### Exploration and alignment of modified regions

Raw sequencing reads are aligned to the human genome using Bowtie2 [43], and duplicates are removed for filtering with SAMtools [44]. Next, the paired reads are normalized and compared to the total read count in BedGraph format, and then converted to bigwig format for further visualization in genome browsers such as the UCSC Genome Browser [45]. Ultimately, potential hydroxymethylated regions (hMRs) are identified using MACS, and peak calling is merged using bedtools merge, retaining only those peaks that appear in more than 10 samples and are less than 1000 bp, during which blacklisted genomic regions showing artifact signals can be filtered out. In this study, hMRs for each patient is generated by intersecting individual peak call files with the combined peak file [46, 47]. Additionally, before conducting differential analysis, genes on the X and Y chromosomes are filtered out.

### Identification of differential regions and related functional enrichment analysis

We conducted differential analysis on the 5hmC regions of 124 patients in cohort 1 (62 cases each of QYDS and NQYDS), using the limma package in R to analyze differential metabolites through a two-sided Wilcoxon rank-sum test (with criteria of |log2 fold change|> 0.4 and *P*-value < 0.05) [48]. Unsupervised hierarchical clustering and heatmap analysis are conducted using the pheatmap package in R. Principal component analysis (PCA) is performed on differential sites using the prcomp package in R. Employing the R “WGCNA” package [49], we performed clustering and module analysis, calculating the module eigengenes, module significance, and their interrelationships. Enrichment analysis is performed using the clusterProfiler package in R, conducting Gene Ontology Biological Process (GOBP) as well as KEGG (Kyoto Encyclopedia of Genes and Genomes) Pathway enrichment analysis [50]. Enrichment analyses were filtered at p < 0.05, with gene-set sizes restricted to 10–500 genes, and only the top-ranked results are presented. The GSEA (Gene Set Enrichment Analysis) enrichment was performed using the clusterProfiler package, with the results adjusted for multiple testing using the False Discovery Rate (FDR) method. For GSEA, the same gene-set size limits were applied, and results were retained if the FDR-adjusted *P*-value was < 0.05, again displaying the top hits. Symptom-gene association data is sourced from the Symmap database [51]. Traditional Chinese medicine-target association data is derived from the HIT 2.0 database [52]. The network graph is created using Cytoscape software [53].

### Selection of feature regions and construction of diagnostic model

We employed 12 feature selection algorithms, including Random Forest, Recursive Feature Elimination with Cross-Validation (RFECV), Logistic Regression, Chi-square Test, Analysis of Variance (ANOVA) F-test, Mutual Information, Least Absolute Shrinkage and Selection Operator (LASSO) Regression, Ridge Regression, Elastic Net, PCA, Linear Discriminant Analysis (LDA), and Autoencoder, to filter feature genes. After a comprehensive evaluation, we selected the top three performing algorithms for an integrated feature selection process. The final feature set consists of genes identified by at least two of these three algorithms. To develop a robust and reproducible polygenic diagnostic model, we devised a nested, repeated cross-validation workflow under an ensemble-learning framework. The pipeline is built around an outer loop and an inner loop. In the outer loop the model is evaluated on the validation set plus Cohort 2, and the entire procedure is repeated 100 times to average out run-to-run variability. Within each outer repetition, the inner loop performs stratified fivefold cross-validation with 10 repeats (50 folds in total) on the training data to train a parameter-free ensemble model. For model construction, we combined three complementary learners—neural networks, random forests, and stochastic gradient descent (SGD)—into an ensemble. Neural networks capture intricate non-linear interactions among features, random forests provide robustness against overfitting while modeling complex decision boundaries, and SGD efficiently minimizes composite-regularized loss functions in an iterative fashion. Probabilistic outputs from these base learners are concatenated into a unified feature matrix, which is then fed to a support-vector machine (SVM) to optimally integrate the individual predictions, yielding the final diagnostic model. The trained model was subsequently applied to both the validation set and Cohort 2. Predictive performance was quantified with the area under the ROC curve (AUC); the optimal cut-off was determined with the ROC-R package in R, from which corresponding specificity and sensitivity were calculated.

### Statistical analysis

All continuous variables are expressed as mean ± (standard deviation) and compared between groups using one-way ANOVA. Non-continuous and categorical variables are presented as frequencies or percentages and compared using the chi-square test. A two-sided *P*-value < 0.05 is considered to indicate statistical significance. Statistical analysis was performed using R.

##  Results

###  Patients’ characteristic

Ultimately, we enrolled a total of 196 patients, with cohort 1 comprising 124 patients (62 with QYDS and 62 with NQYDS), and cohort 2 consisting of 72 patients (37 with QYDS and 35 with NQYDS). We conducted statistical analyses and comparisons on various parameters including age, body weight, height, BMI, SBP, DBP, HR, cardiac-related questionnaires, hypertension, COPD, type 2 diabetes, obstructive sleep apnea, hyperlipidemia, cerebral infarction, old myocardial infarction, chronic heart failure, arteriosclerosis, history of radiofrequency ablation, history of percutaneous coronary intervention (PCI), history of coronary artery bypass grafting, pacemaker status, and biochemical markers such as Glucose, Urea, Creatinine, Total cholesterol, Triglycerides, High-density lipoprotein cholesterol (HDL-C), and Low-density lipoprotein cholesterol (LDL-C). The results showed no significant differences in these indices between QYDS and NQYDS patients in either cohort 1 or cohort 2, indicating that baseline characteristics were well balanced between the QYDS and NQYDS groups in both cohorts and thus minimizing the potential for confounding (Table 1).

**Table 1:**
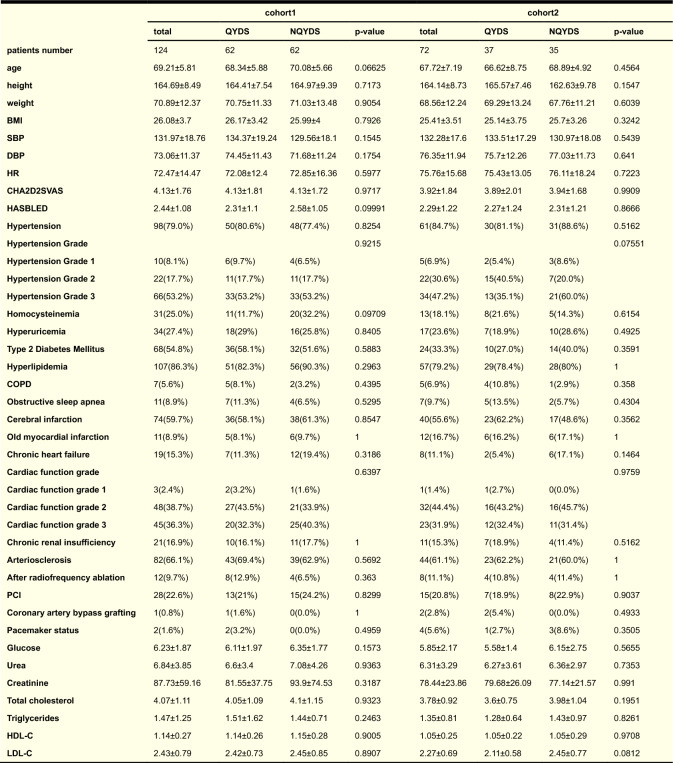
The summary of basic characteristics and clinically relevant indices for all participating patients in this study

The table presents the baseline survey data of the patients, where count data is displayed in the form of numbers (percentage), and measurement data is presented as the mean ± standard deviation. *P*-values were derived after selecting the optimal test—two-sample t-test, Pearson’s Chi-squared, Fisher’s exact, Wilcoxon rank-sum, or Kruskal–Wallis—based on data type, sample size, and the results of normality and variance homogeneity checks. Abbreviations: BMI, Body Mass Index; SBP, Systolic Blood Pressure; DBP, Diastolic Blood Pressure; HR, Heart Rate; CHA2D2SVAS, an acronym for Congestive Heart Failure, Hypertension, Age ≥ 75 (doubled), Diabetes, Stroke (doubled), Vascular Disease, Age 65–74, and Sex Category (female); HASBLED, the bleeding risk score for anticoagulant therapy in atrial fibrillation; COPD, Chronic Obstructive Pulmonary Disease; PCI, Percutaneous Coronary Intervention; HDL-C, High-Density Lipoprotein Cholesterol; LDL-C, Low-Density Lipoprotein Cholesterol

### The genome-wide 5hmC profiles of evDNAs differ between the QYDS and NQYDS patients

**Fig. 2:**
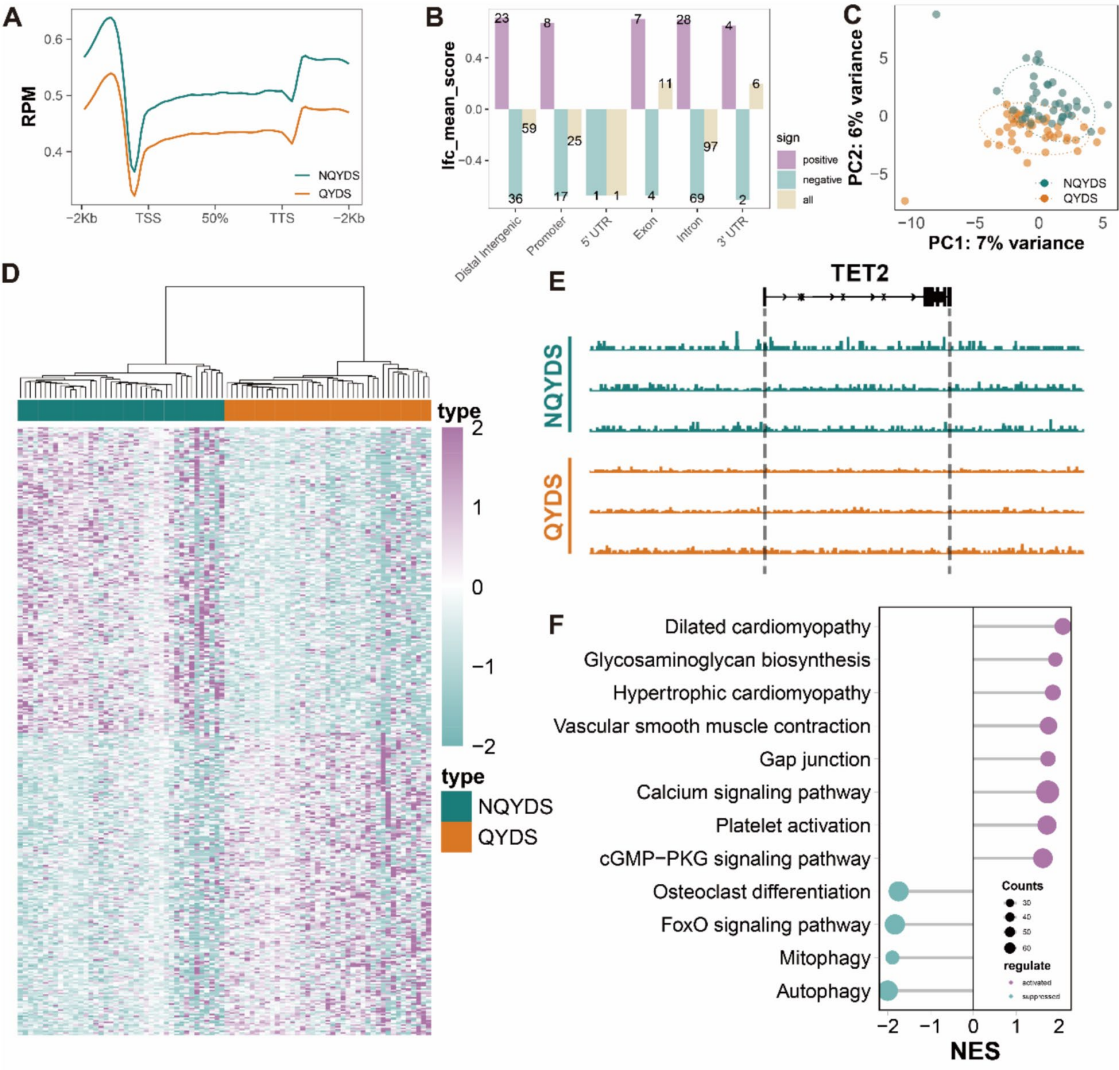
The genome-wide 5hmC profiles of evDNAs differs between the QYDS and NQYDS groups. **A** The genome-wide 5hmC distribution characteristic profiles of the QYDS and NQYDS groups. **B** The gene distribution map of markers identified through 5hmC-Seq analysis. **C** The PCA plot clearly distinguishes between the two groups, QYDS and NQYDS. **D** The heatmap reflects the expression of the top 200 DhMRs in each sample of QYDS and NQYDS, with red indicating upregulation and blue indicating downregulation. **E** The genomic browser view of TET2 in plasma evDNAs samples from QYDS and NQYDS groups. **F** The GSEA lollipop plots for QYDS and NQYDS

To comprehensively understand whether there are differences in the whole-genome 5hmC profiles of plasma evDNAs between the QYDS and NQYDS groups, we first compared the distribution of 5hmC along the genomes of the two groups and found that the 5hmC modification levels in patients with QYDS are significantly lower than those in NQYDS and healthy individuals (Fig. 2A). Next, we analyzed the distribution of 5hmC markers across different genomic feature regions between groups and found that 5hmC markers were predominantly located in distal intergenic, intron regions, and promoter (Fig. 2B). The results are consistent with previous studies on 5hmC in cardiovascular diseases, and additionally, most 5hmC in mammals is enriched in intragenic, intron and promoter regions [17, 18]. And indicating a close relationship between 5hmC and gene expression, thereby validating the reliability of the 5hmC-Seal method [54].

Next, to investigate the changes in 5hmC signals between the QYDS and NQYDS groups in AF patients, we first performed PCA on genes with differentially regulated 5hmC levels and found that QYDS and NQYDS could be distinguished in the PCA plot (Fig. 2C). Then, we performed unsupervised hierarchical clustering on the top 400 differentially hydroxymethylated regions (DhMRs) (200 upregulated and 200 downregulated) detected from the QYDS and NQYDS groups. The resulting heatmap clearly distinguishes the samples from the two groups (Fig. 2D). Thus, these findings indicate that DhMRs from evDNAs may possess the capability to differentiate between AF patients with QYDS and those with NQYDS. Recent studies have identified TET2 as a key regulator of 5hmC and highly associated with increased risk of AF [8, 55, 56]. The genomic browser plots reveal that in both intragenic and perigenic regions, the 5hmC peak signal levels of TET2 in QYDS are significantly lower than those in NQYDS individuals, with NQYDS showing slightly higher levels than QYDS (Fig. 2E). Our findings reveal differential 5hmC expression levels of TET2 between QYDS and NQYDS, suggesting the potential of plasma evDNAs 5hmC to distinguish between these two conditions. Furthermore, we conducted a GSEA enrichment analysis on the overall differentially 5hmC-modified genes (DhMGs) to observe the changes in global pathways (Fig. 2F and Supplementary material S2). The results indicate that compared to NQYDS, QYDS exhibits upregulation in pathways associated with cardiac-related diseases such as Dilated Cardiomyopathy and Hypertrophic Cardiomyopathy. Additionally, the Calcium Signaling Pathway, which is related to AF [57], is upregulated in QYDS. This suggests that the QYDS subtype of AF may be more inclined towards alterations in cardiac morphology and calcium ion signaling pathways.

### WGCNA and enrichment analysis uncovered the network modules and associated functions of 5hmC modified genes

**Fig. 3:**
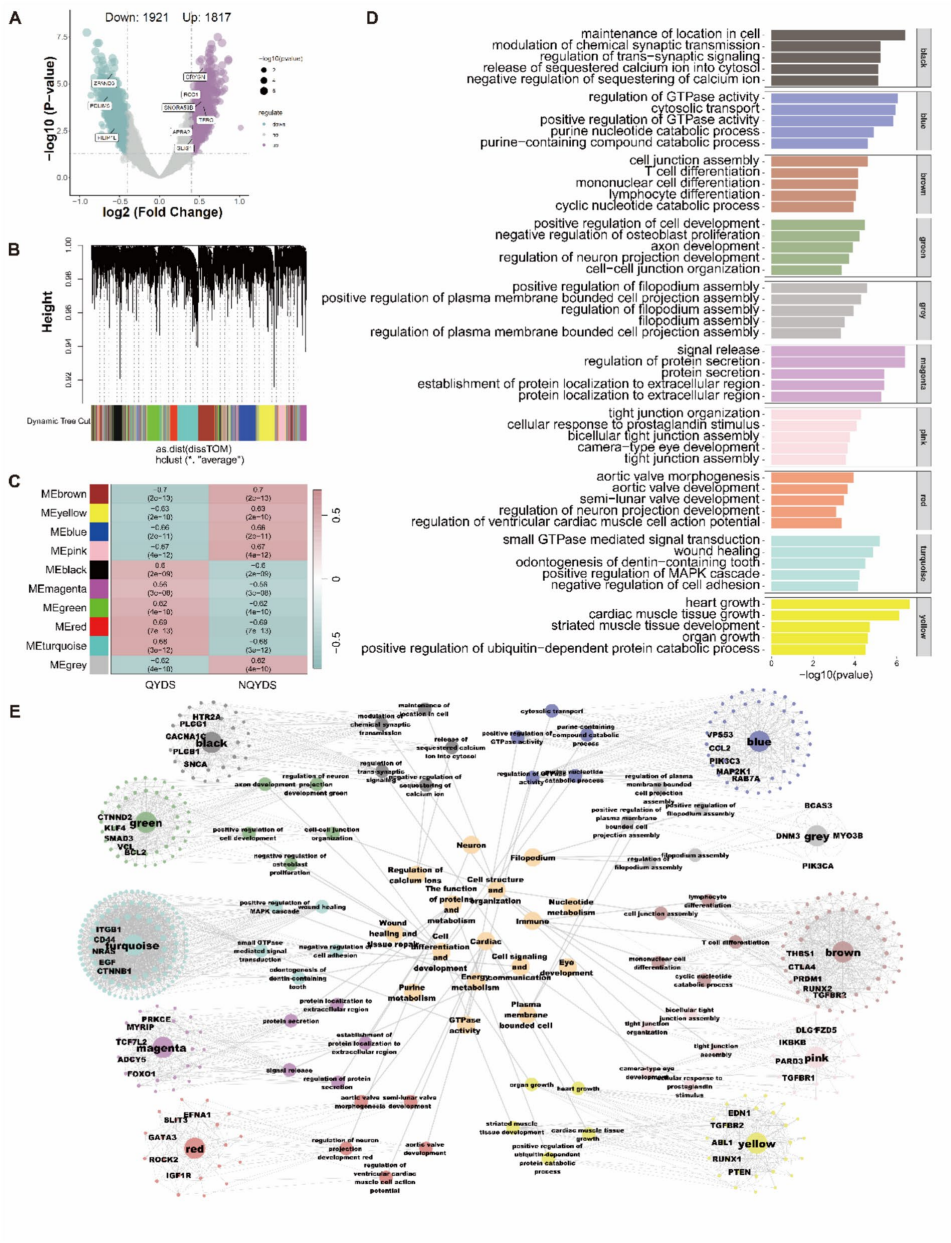
WGCNA, associated functional enrichment analysis, and network construction. **A** The volcano plot, with upregulated differential sites marked in purple and downregulated sites in green, where the size of the points correlates with the *P*-value. **B** The dendrogram display from the WGCNA. **C** A heatmap illustrating the correlation of each module with QYDS and non-QYDS, with *P*-values indicated in parentheses. **D** The GO enrichment analysis results for various color-coded modules, showcasing the top 5 results with the highest -log10 (*P*-value) ranking within each module. **E** The molecular network features of the modules from the WGCNA analysis, complemented by the corresponding functional enrichment results and annotations. These molecular network characteristics are derived from the PPI network, and each network is annotated with the top 5 genes ranked by degree value within that module

Subsequently, using the DESeq2 package, we identified DhMGs in all AF patients (p < 0.05, |log2 fold change|> 0.4). Compared to the NQYDS group, we detected 1817 upregulated DhMGs and 1921 downregulated DhMGs (Fig. 3A and Supplementary material S3). To further investigate the differences between QYDS and NQYDS, we conducted Weighted Gene Co-expression Network Analysis (WGCNA) analysis on DhMGs, resulting in the construction of 10 molecular modules (Fig. 3B). Among them, five modules were negatively correlated with QYDS, ranked by relevance as brown, pink, blue, yellow, and grey. Conversely, five modules were positively correlated with QYDS, ranked by relevance as red, turquoise, green, black, and magenta (Fig. 3C). Subsequently, to elucidate the functions of the distinct molecular modules, we conducted an enrichment analysis of Biological Processes (Fig. 3D). The results revealed that the black module is correlated with the maintenance of intracellular localization, cellular signal transduction, and regulation of calcium ions. The blue module is associated with GTPase activity and purine metabolism. The brown module is linked to the differentiation of immune cells. The green module is related to the development of neuronal cells and the proliferation of osteoblasts. The grey module is involved in filopodium assembly. The magenta module is connected to protein secretion and localization. The pink module is related to tight junctions and prostaglandins. The red module is associated with the morphology and development of the aortic valve, as well as the action potentials of cardiomyocytes. The turquoise module is involved in GTPase signal transduction, wound healing, the MAPK signaling pathway, and cell adhesion. The yellow module correlates cardiac development, myocardial development, striated muscle development, and protein metabolism. Based on the aforementioned findings, we constructed a WGCNA module-5hmC differential site-function network diagram (Fig. 3E), annotating genes related to differential sites with high degree values within each module, such as *TGFBR2*, *RUNX2*, *PRDM1*, *CTLA4*, and *THBS1* in the brown module.

QYDS is a traditional Chinese medicine diagnosis that encompasses both Qi deficiency and Yin deficiency. Qi deficiency is primarily characterized by symptoms such as pale complexion, shortness of breath, weakness in the limbs, dizziness, excessive sweating, and a slow pulse [58, 59]. It is often associated with decreased metabolic energy, reduced blood circulation efficiency, lowered muscle excitability, weakened metabolic processes, and aging [60, 61]. Yin deficiency in TCM manifests clinically as a set of symptoms indicating impaired water and energy metabolism, including dry eyes, dry mouth, dry skin, a warm sensation in the palms and soles, facial redness, and sweating [62, 63]. Previous studies have shown that it is primarily associated with the activity of the immune system, regulation of inflammation, energy metabolism, and endocrine regulation [64, 65]. These research findings are similar to ours. Furthermore, because our study employs a more sensitive epigenetic sequencing method, we have identified some functions that have not been previously reported and may be associated with QYDS, such as GTPase activity, purine metabolism, heart development, neuronal cell development, protein secretion and localization, etc. These features will require further validation in the future to explore their relationship with QYDS.

Notably, the WGCNA network analysis has identified a substantial correlation between the red module and both the developmental and morphological aspects of cardiac valves, as well as the electrical potential of cardiomyocytes, which exhibits a positive correlation with the progression of QYDS. Extant research has highlighted the intimate link between alterations in cardiac valve morphology, interventions that impact valve structure, and the genesis of AF [66, 67]. Furthermore, modifications within modules associated with the cardiac valve pathway at the epigenetic level in QYDS underscore the necessity for close surveillance of cardiac valve dynamics during the management of AF patients afflicted with QYDS. In our study, the red module was found to be closely associated with the morphology and growth of cardiac valves, involving genes such as *IGF1R*, *ROCK2*, and *GATA5*. Studies have indicated that *IGF1R* can offer protection against AF independently of the PI3K-Akt pathway and *HSP70* [68]. Additionally, research suggests that the absence of *ROCK2* expression can lead to the occurrence of intracardiac arrhythmias in AF [69]. The study also found that *GATA5* gene mutations are independently associated with AF. Our data demonstrate a positive correlation between *IGF1R, ROCK2* and *GATA5* with QYDS, highlighting the potential importance of these genes’ expression in the QYDS subtype of AF treatment. WGCNA analysis also suggests a negative correlation between immune-related pathways and QYDS, with the brown module implicating multiple immune-related pathways such as monocyte differentiation, lymphocyte differentiation, and T-cell differentiation. Immunity is intimately involved in AF pathogenesis. Pre-clinical studies demonstrate that blocking monocyte migration attenuates AF in mice, with macrophages acting as pivotal mediators of immune-modulatory therapy [70]. Cytotoxic T-lymphocytes in the atrial epicardium can remodel the substrate, thereby promoting the occurrence of AF [71]. Consistently, clinical and experimental reports indicate that limiting immune infiltration and down-regulating pro-inflammatory cytokines can ameliorate AF [72, 73]. This may indicate that AF patients with NQYDS need to pay more attention to the application of immunosuppressive drugs. The gene with the highest degree value in the brown module is *TGFBR2*. Studies have shown that reduced expression of *TGFBR2* can mitigate fibrosis, which in turn aids in the beneficial remodeling of atrial conduction and recovery properties in the left atrium, thereby treating AF [74]. Our research indicates a negative correlation between QYDS and *TGFBR2*, suggesting that the NQYDS subtype of AF treatment may require closer attention to the expression of the *TGFBR2* gene. These analyses have successfully unveiled the epigenetic molecular basis of TCM syndrome differentiation, thereby laying the groundwork for the implementation of objective and nuanced epigenetic diagnosis and treatment (Epi-DT).

### Establishing connections between symptom-pathway networks and drug-pathway networks with 5hmC modified genes

**Fig. 4:**
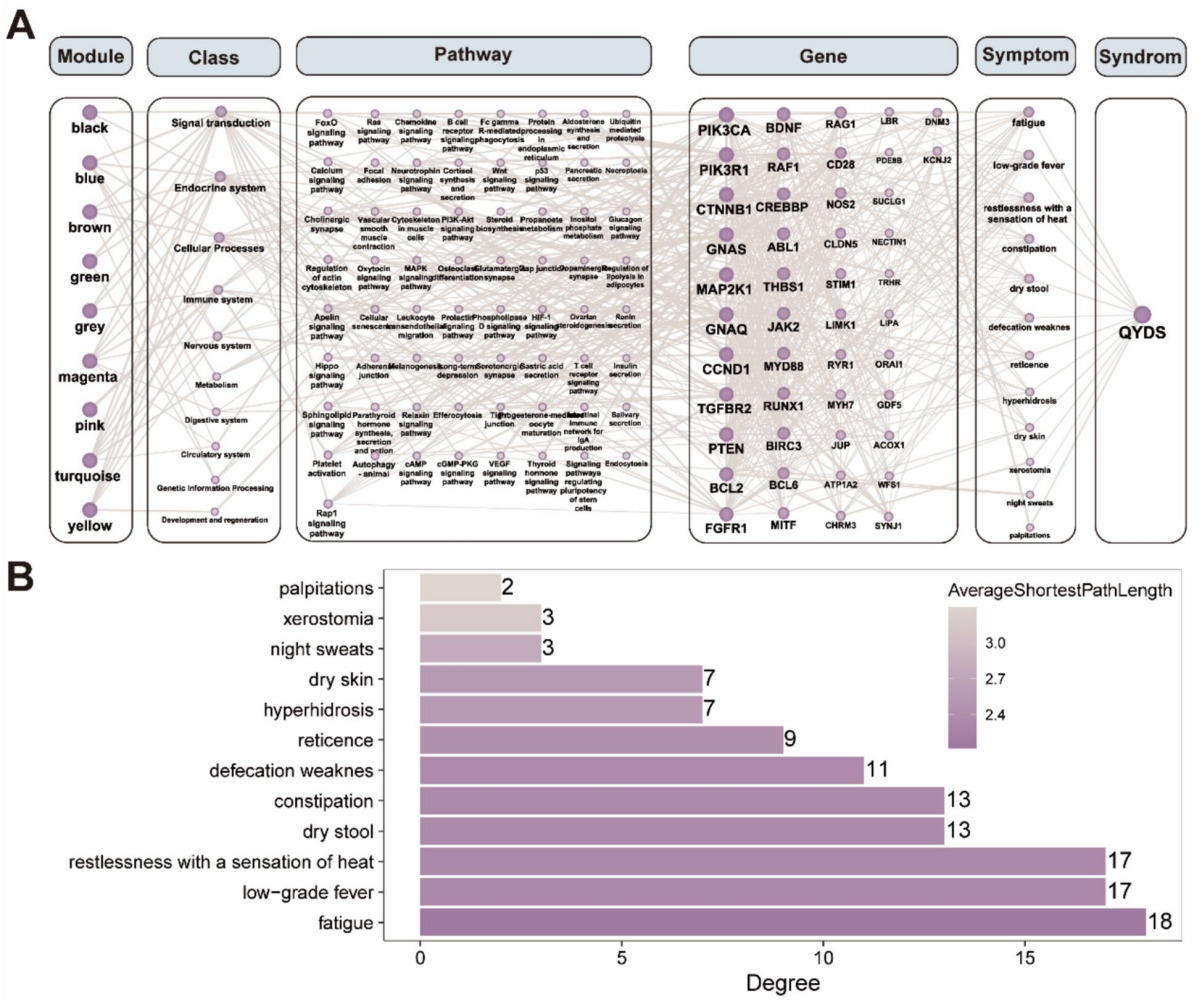
Construction and Analysis of a Module-Class-Pathway-Gene-Symptom-Syndrome Network Diagram. **A** The network diagram of Module-**Class**-Pathway-Gene-Symptom-Syndrome, constructed by integrating symptom data from the Symmap database with differential 5hmC regions, where the size of the nodes is positively correlated with their degree values. **B** The horizontal axis represents the degree values of various symptoms in the network diagram, with the color of the bars indicating the average shortest path length

To further investigate whether there is a connection between 5hmC sites and modules and symptoms, we utilized data from the Symmap database to identify the association between genes related to DhMGs and symptoms. Based on this, we constructed a Module-**Class**-Pathway-Gene-Symptom-Syndrome Network for further analysis. We constructed a network diagram with a total of 143 nodes and 825 edges (Fig. 4A). Topological analysis revealed that the DhMGs with the highest rankings include *PIK3CA*, *PIK3R1*, *CTNNB1*, *GNAS*, and *MAP2K1*, which are primarily associated with signal transduction, the endocrine system, cellular processes, the immune system, and the nervous system. Moreover, these genes are closely related to symptoms associated with QYDS, such as fatigue, low-grade fever, restlessness accompanied by a sensation of heat, dry stool, and constipation (Fig. 4B).

**Fig. 5:**
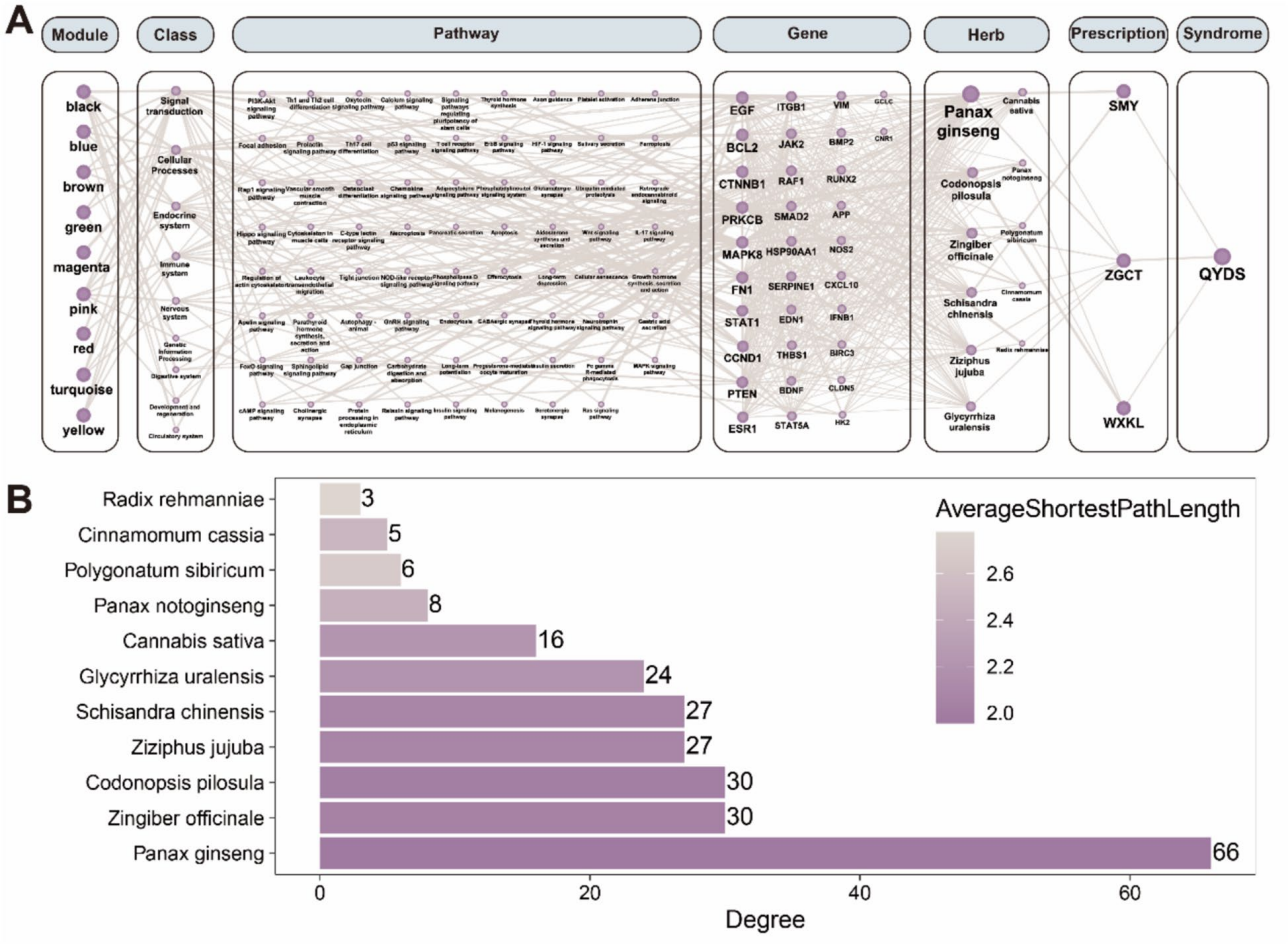
Construction and Analysis of a Module-Class-Pathway-Gene-Herb-Prescription-Syndrome Network Diagram. **A** The network diagram of Module-**Class**-Pathway-Gene-Herb-**Prescription** -Syndrome, constructed by integrating symptom data from the HIT 2.0 database with differential 5hmC regions, where the size of the nodes is positively correlated with their degree values. **B** The horizontal axis represents the degree values of various Chinese herbs in the network diagram, with the color of the bars indicating the average shortest path length

Next, we utilized the HIT2.0 database to collect targets of three commonly used clinical prescriptions for treating cardiopulmonary QYDS syndromes: Shengmai Drink, Roasted Licorice Decoction, and Wenxin Granules. This was done to observe the relationship between DhMGs and existing drugs. Ultimately, we constructed a network with a total of 136 nodes and 710 edges (Fig. 5A). Topological analysis indicated that the DhMGs with the highest rankings are *EGF*, *BCL2*, *CTNNB1*, *PRKCB*, and *MAPK8*, which are mainly associated with signal transduction, cellular processes, the endocrine system, the immune system, and the nervous system. These genes are closely linked to traditional Chinese medicines such as Panax ginseng, Zingiber officinale, Codonopsis pilosula, Ziziphus jujuba, and Schisandra chinensis (Fig. 5B). These findings indicate that targets related to the symptoms of QYDS, as well as drug-related targets, exhibit significant differential expression at the 5hmC level. This discrepancy is further supported by the association between genes implicated in QYDS-related symptoms and medications and their connections to immune function. This corroborates the reliability of the DhMGs identified by the 5hmC-Seal. Therefore, the DhMGs identified through 5hmC-Seal hold promise as potential biomarkers for distinguishing AF patients with QYDS from those with NQYDS.

### Biomarker screening and model construction for QYDS in AF

**Fig. 6:**
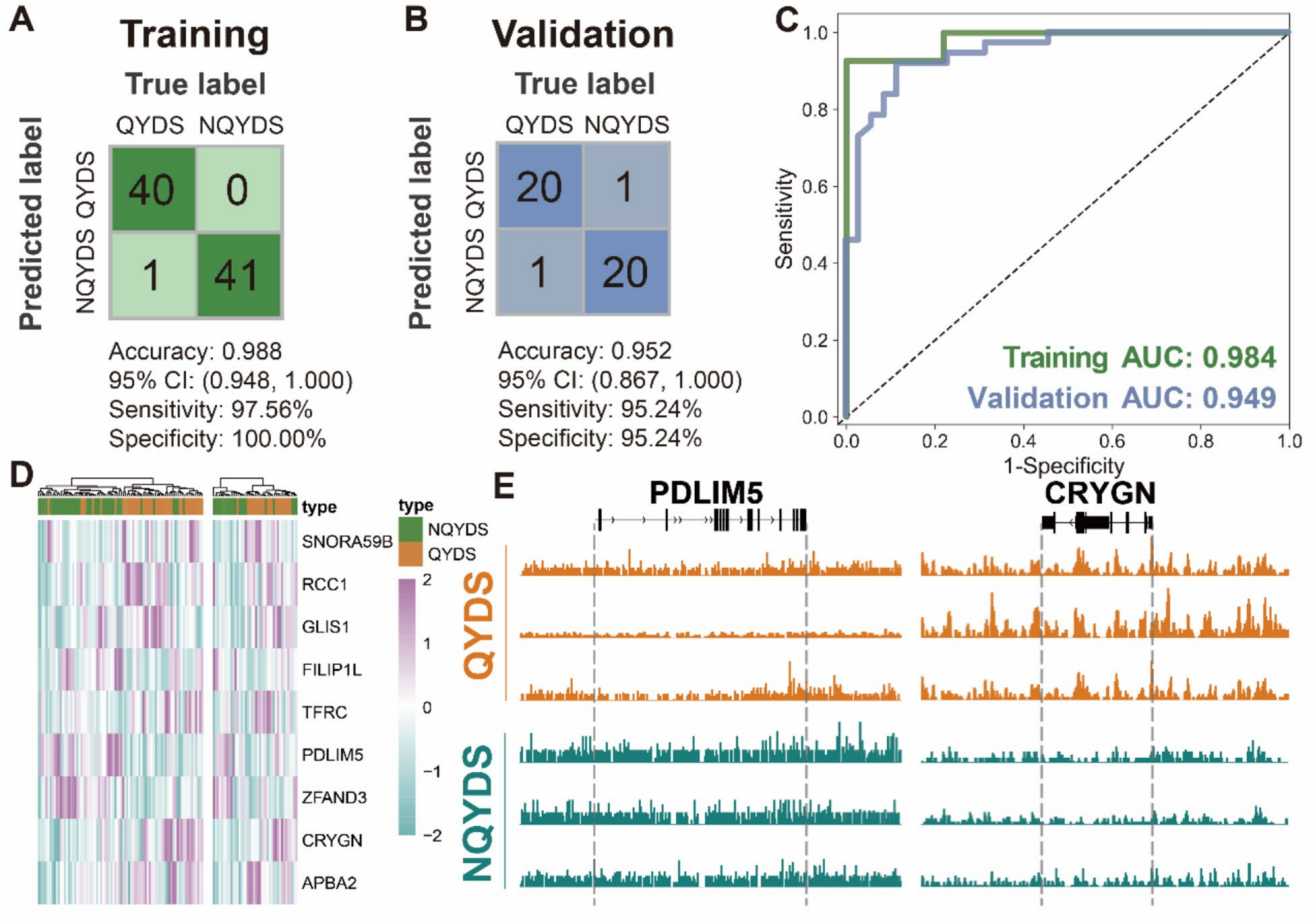
Construction of Model for Distinguishing QYDS and NQYDS in AF Patients through evDNAs in Cohort 1. **A** Process flowchart for model efficacy assessment in the training. **B** Process flowchart for model efficacy assessment in the validation. Partial results from Model Evaluation in Cohort 1, with identified sample numbers indicated in each box. **C** ROC curves for the model's performance in the both sets. **D** Gene expression heatmap for the model on the both set. **E** The genomic browser view for the top-ranked upregulated and downregulated genes based on fold change

To further ascertain whether the 5hmC differentially associated genes of evDNAs can serve as biomarkers to differentiate between QYDS and NQYDS in AF, we divided cohort 1 into a training (n = 82) and a validation (n = 42) in a 2:1 ratio for subsequent analysis. We initially applied 12 machine learning methods to feature selection on DhMGs related to genes in the training (|log2FoldChange|> 0.4, p < 0.05, Supplementary Material S4), combined with correlation analysis to select the most promising feature selection methods. Ultimately, we utilized the top three methods in terms of accuracy values in both the training and the validation, which also showed the weakest correlation in the correlation analysis, namely LDA, logistic regression, and Random Forest (Supplementary Material S4A and B). Subsequently, we used these three algorithms to select the top 80 features for each, and the features finally included in the diagnostic model were the DhMGs that were selected by at least two of these three methods (Supplementary Material S4C). We identified that the 9 DhMGs selected by the model in the training (Supplementary material S5) could effectively distinguish between patients with QYDS and those without NQYDS in AF within the training (Fig. 6A, C). The model demonstrated a high accuracy of 0.988 in the training cohort (95% CI 0.948–1.000). Furthermore, the heatmap revealed that the 9 DhMRs could effectively differentiate between the two TCM syndrome types of AF patients (Fig. 6D). Subsequently, we validated the model using the validation, achieving a high level of accuracy (Fig. 6B, C). The validation exhibited an AUC of 0.952 (95% CI 0.867–1.000). The heatmap analysis further confirmed that the model could still effectively distinguish between the two TCM syndrome types of AF patients (Fig. 6D). The genes implicated in our model, such as *SNORA59B*, *RCC1*, *GLIS1*, *FILIP1L*, *TFRC*, *PDLIM5*, *ZFAND3*, *CRYGN*, and *APBA2* are predominantly associated with protein processing, transcription regulation, immunity, as well as cell cycle, cell apoptosis, ferroptosis, cytoskeleton, crystalline lens, nervous system [75–82], which parallels the diagnostic criteria for QYDS and NQYDS. Additionally, we visualized the most significantly up- and down-regulated DhMGs in a genomic-browser view, revealing a marked divergence in the 5hmC signals of PDLIM5 and CRYGN between QYDS and NQYDS (Fig. 6E). Corresponding box plots for the nine signature genes in the training cohort confirm consistent, significant inter-group differences (Supplementary Figure S6). Furthermore, we have generated ROC curves for each of the differential sites, demonstrating that each individual differential region-associated gene possesses good diagnostic capability, with AUC values all exceeding 0.7 (Supplementary material S7). Additional information regarding the diagnostic model can be found in Supplementary Material S8. These findings suggest that the DhMRs identified by 5hmC-Seal can be utilized to construct a highly accurate diagnostic model, potentially leading to the development of promising molecular biomarkers.

### Performance of the diagnostic model in an independent double-blind cohort

**Fig. 7:**
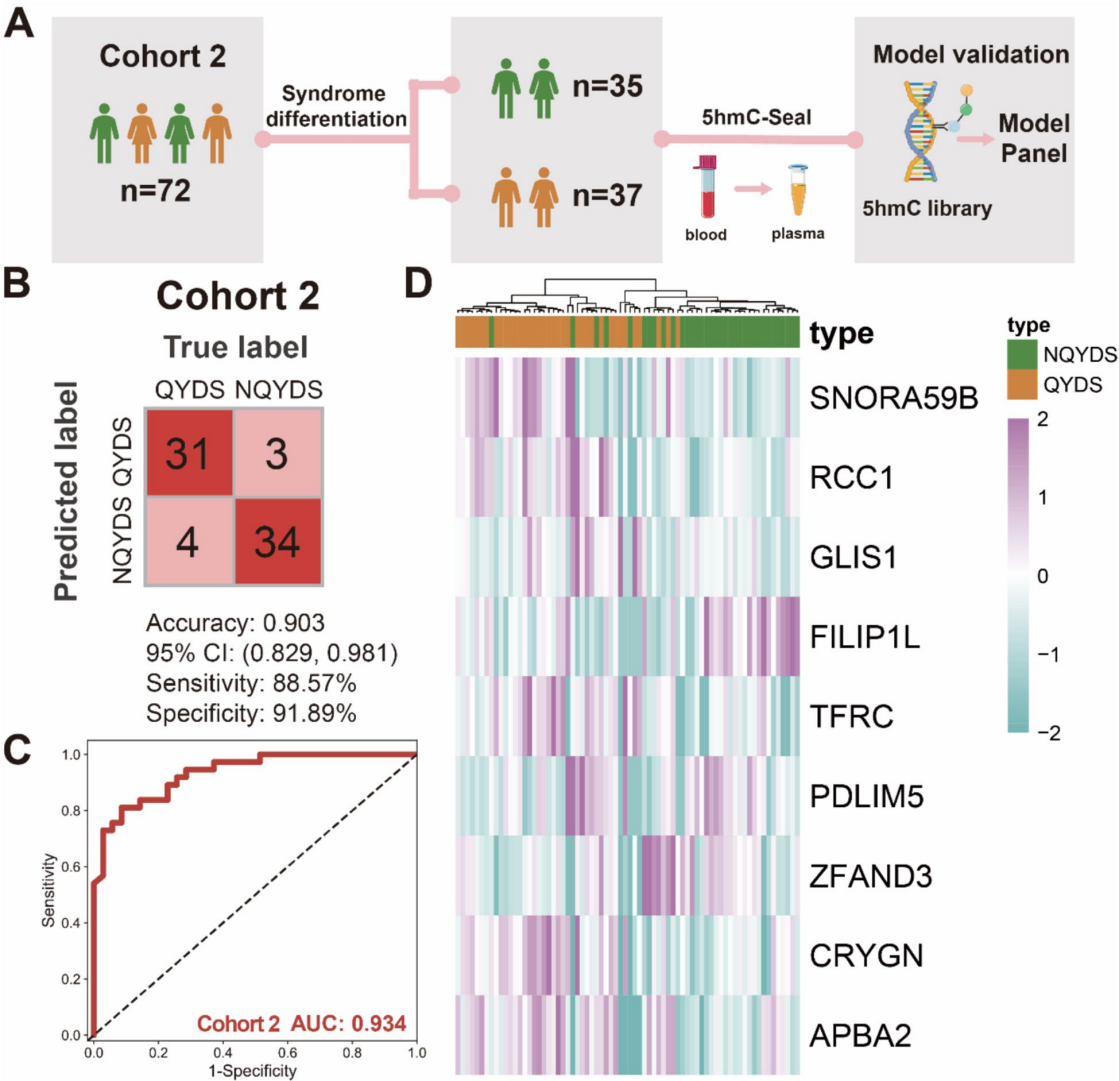
Validation of Model for Distinguishing QYDS and NQYDS in AF Patients through evDNAs in Cohort 2. **A** Process flowchart for model efficacy assessment in Cohort 2. **B** Partial results from Model Evaluation in Cohort 2, with identified sample numbers indicated in each box. **C** ROC curves for the model's performance in the cohort 2. **D** Gene expression heatmap for the model on the cohort 2

To further validate the reliability of the model, we collected an independent cohort of AF patients separate from Cohort 1, to test the model constructed using the 5hmC DhMGs associated with genes from Cohort 1. We enrolled a total of 72 patients, comprising 35 individuals with QYDS and 37 with NQYDS. We similarly extracted evDNAs using the aforementioned method to perform the 5hmC-Seal analysis (Fig. 7A). We observed that within Cohort 2, the model continued to effectively differentiate between AF patients with QYDS and those without NQYDS (Fig. 7B, C). The model demonstrated an accuracy of 0.903 (95% CI 0.829–0.981) in the cohort 2. The heatmap revealed that the 9 DhMGs associated with genes can effectively distinguish between the two TCM syndrome types of AF patients (Fig. 7D). Additionally, we compared the ROC curves of each individual algorithm on the external validation and found that the model constructed through the integration of the three algorithms performed better than the models built by individual algorithms alone (Supplementary material S5D). Finally, based on the GO analysis results from WGCNA, we created a Sankey diagram for these 9 DhMGs to understand their associated functions (Supplementary material S9). In addition, we conducted correlation analyses to observe the relationships between the nine DhMGs and clinical characteristics. We found that these nine DhMGs were correlated with DBP, CHA2DS2-VASc, HAS-BLED, Classification of Hypertension, Urea, Total cholesterol, and LDL-C (Supplementary material S10). This result indicates that the model constructed from evDNAs based on 5hmC-Seal can withstand validation by external cohorts, demonstrating a certain level of reliability in the model.

### Diagnostic model combining biomarkers with clinical symptoms

**Fig. 8:**
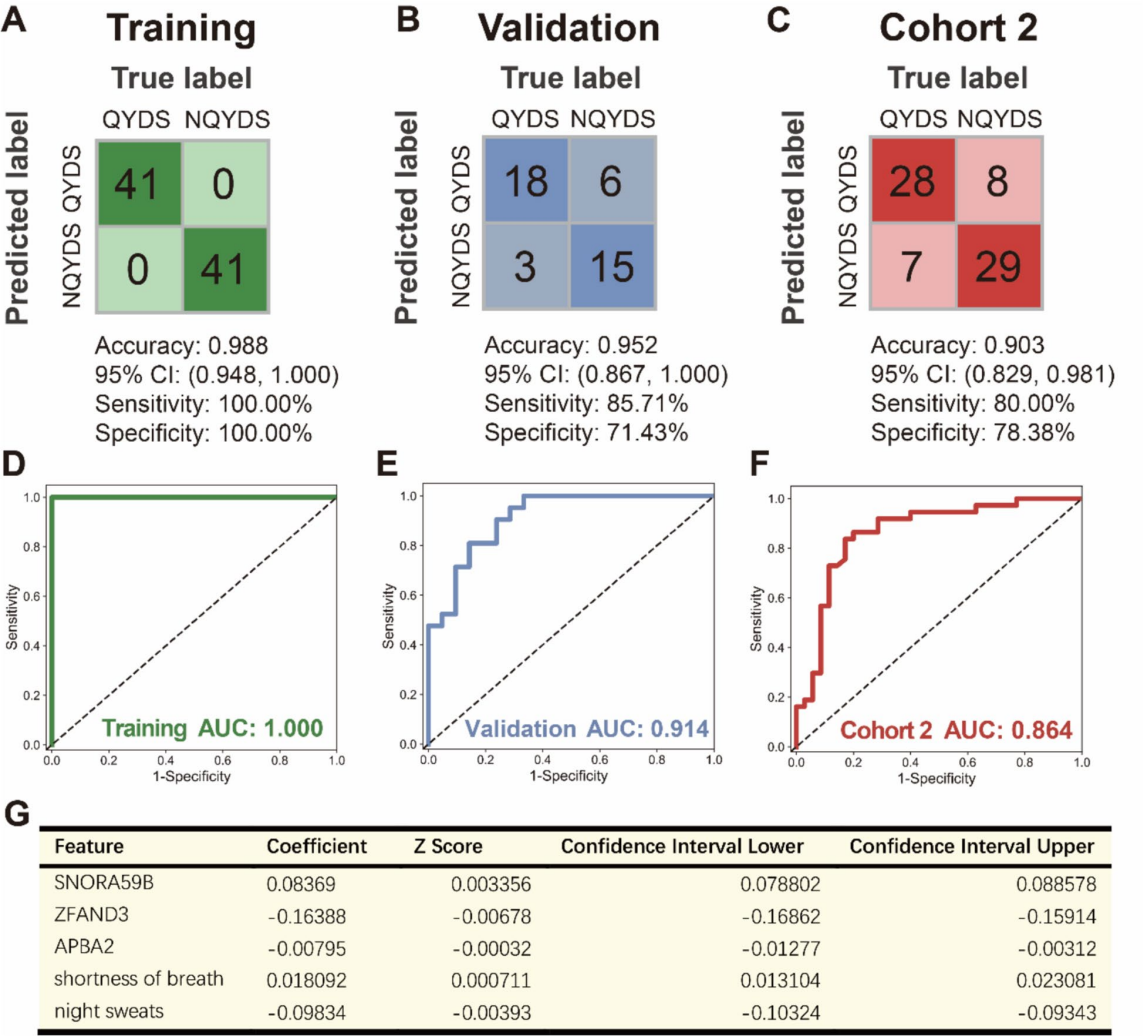
Diagnostic Model Combining Biomarkers with Clinical Symptoms. **A**–**C** The AUC curves of the diagnostic model combining biomarkers with clinical symptoms in the training group, the control group, and cohort 2. **D**–**F** ROC curves for the model's performance in sets. **G** Table shows the relevant parameters in the diagnostic model combining biomarkers with clinical symptoms

To reduce the number of biomarkers while enhancing accuracy, we integrated symptoms and molecular markers to comprehensively reflect disease characteristics, compensating for the limitations of single diagnostic criteria. This approach improves diagnostic accuracy and reliability. We converted symptoms into binary variables and combined them with 9 DhMGs, using previous screening and modeling methods to reselect markers and build diagnostic models. Ultimately, we identified a diagnostic model comprising 3 DhMGs (SNORA59B, ZFAND3, APBA2) and two symptoms (shortness of breath and night sweats). The model achieved an accuracy of 1.000 (95% CI 0.968–1.000) in the training set, 0.786 (95% CI 0.661–0.929) in the test set, and 0.792 (95% CI 0.699–0.896) in cohort 2 (Fig. 8A, C), with high AUC values of 1.000, 0.914, and 0.864, respectively (Fig. 8D, F). Specific model parameters are shown in Fig. 8G. In summary, these results suggest that combining traditional symptoms with modern epigenetic molecular markers can reduce the number of molecular markers while maintaining accuracy, potentially providing a basis for modern integrated traditional Chinese and Western medicine diagnosis.

## Discussion

Epigenetics focuses on how gene expression is regulated without changes in the genetic sequence. As research progresses, it has been found that the holistic, dynamic, individual, and interactive characteristics of epigenetics with the environment are highly consistent with the core concepts of TCM syndrome typing [9, 10, 83]. TCM syndrome typing reflects the pathophysiological phase resulting from the interaction of internal and external environments with the human body, which can regulate the manifestation of symptoms and thus affect the occurrence and development of complex diseases. This perspective aligns with the reversible regulatory concept of epigenetics. Currently, a study has conducted bisulfite sequencing on patients with different TCM syndromes of endometriosis and found that the changes in DNA methylation in peripheral blood samples may be associated with TCM syndrome types [84].

Therefore, studying the connection between epigenetics and TCM syndrome typing is very important.

Emerging research has underscored the critical function of 5hmC in modulating gene expression, establishing its significance for the discovery and validation of biomarkers pivotal to disease diagnosis and prognostic assessment [16, 20, 21, 42]. 5hmC is not merely a fleeting intermediate in the 5mC oxidation pathway; it is an autonomous, stable, and functionally rich epigenetic signal in its own right. Recent studies show that 5hmC marks active genomic regions—so-called “epigenetically dynamic loci”—and is especially enriched at cell-type-specific enhancers, where it modulates the expression of transcription-factor-bound genes [85]. Owing to its high stability and sensitivity, 5hmC captures genome-wide epigenetic information and encodes quantitative insights into gene-regulatory networks [86, 87]. These attributes have established 5hmC as a powerful biomarker for the discovery and validation of disease associations. Similarly, recent research has also discovered that evDNAs in the blood is instrumental in disease diagnosis and therapeutic efficacy monitoring [32, 33]. This study innovatively integrates the 5hmC-Seal technology with evDNAs to explore the epigenetic molecular characteristics and biomarkers of TCM syndromes in QYDS, and to construct a molecular diagnostic model. Our study innovatively proposes Epi-DS as an objective basis for the fundamental research of TCM syndrome typing, offering a new perspective for further investigation into molecular mechanisms and contributing to the modernization and internationalization of traditional Chinese medicine.

This study takes the hydroxymethylation data of AF with QYDS and NQYDS as an example to illustrate the role of Epi-DS in the diagnosis of TCM syndromes. By comparing the hydroxymethylation profiles of plasma samples between QYDS and NQYDS, we found that the hydroxymethylation level of patients with QYDS was significantly lower than that of NQYDS. Next, results of the heatmap and PCA plot indicate that there are distinct hydroxymethylation distribution characteristics between patients with QYDS and those NQYDS, and that 5hmC markers can effectively differentiate between QYDS and NQYDS patients. These findings provide a detailed molecular portrait of the epigenetic landscape associated with QYDS in AF patients, highlighting the potential role of 5hmC in TCM syndrome.

Furthermore, in order to analyze the hydroxymethylation profile characteristic of patients with QYDS, compared to those NQYDS, we found that there are 1817 upregulated DhMGs and 1921 downregulated DhMGs in QYDS patients. Meanwhile, we employed WGCNA to explore the functional analysis of genes associated with DhMGs identified by 5hmC. Our study identified a total of 10 modules closely related to QYDS, involving multiple pathways and functions. Notably, we identified that the two modules most closely associated with QYDS—the red module (positively correlated) and the brown module (negatively correlated)—respectively relate to cardiac valvular development and morphology, as well as various immune functions such as monocyte differentiation, lymphocyte differentiation, and T-cell differentiation (Fig. 3D). This indicates that patients with QYDS and NQYDS subtypes constitute distinct molecular populations. Our findings successfully elucidate the epigenetic molecular basis of TCM syndrome differentiation, substantiating the rationality of TCM’s diagnostic classification of AF at the molecular level and laying the foundation for objective and detailed Epi-DT. In summary, our study has identified distinct molecular and functional features of 5hmC in QYDS compared to NQYDS. For example, our results show significant differences in immune cell differentiation and the energy metabolism pathway, specifically the MAPK signaling pathway. These features also reflect key characteristics of QYDS in traditional Chinese medicine theory, including imbalances in energy metabolism and relative weakening of immune capacity. This part illustrates the specificity of 5hmC in QYDS.

Furthermore, to delve deeper into the differential targets identified by 5hmC-Seal, we constructed the symptom and drug networks to observe the connections between DhMGs and symptoms and drugs. In our study, we found that the DhMGs have connections with many clinical symptoms associated with QYDS, such as fatigue, low-grade fever, restlessness accompanied by a sensation of heat, dry stool, and constipation. According to the theoretical system of TCM, these related symptoms are highly closely related to QYDS [58, 59, 62, 63]. Additionally, we discovered that many DhMGs are closely related to QYDS related medications, particularly Panax ginseng, Zingiber officinale, Codonopsis pilosula, Ziziphus jujuba, and Schisandra chinensis. Modern research has also found that Panax ginseng, Codonopsis pilosula, Ziziphus jujuba, and Schisandra chinensis are common traditional Chinese medicinal herbs used to treat QYDS. They have regulatory effects on various biological functions such as signal transduction, cellular processes, the endocrine system, and the immune system [88–91]. It is noteworthy that both the symptoms and medications associated with QYDS suggest an immune-related etiology. Consequently, alterations in immune status may represent a promising avenue for future research into QYDS. Ultimately, we found that our research results could be corroborated with existing databases to find relevance to symptoms associated with QYDS and clinically used drugs for QYDS. This further demonstrates the reliability of 5hmC-Seal in plasma evDNAs, indicating that the differential region-related genes identified by this method can serve as biomarkers for further diagnostic model construction.

Subsequently, we employed a variety of feature selection algorithms and integrated multiple modeling techniques to model the DhMRs identified by 5hmC-Seal in the training cohort. To prevent overfitting and enhance the reliability of the constructed model, we utilized an independent double-blind validation cohort for verification. After multiple comparisons, we identified a model panel composed of 9 DhMGs identified by 5hmC-Seal, which demonstrated high accuracy in the training (AUC = 0.984), validation (AUC = 0.949), and external cohorts (AUC = 0.934). Notably, our optimized integrated algorithm not only avoided overfitting but also achieved high diagnostic efficacy in the external independent validation cohort compared to individual algorithms. Compared to previous studies on molecular diagnostic models for TCM syndromes, our epigenetic sequencing based on 5hmC-Seal has significantly improved accuracy. The accuracy of our model in the training, validation, and external double-blind validation cohorts are markedly higher than that of previous diagnostic model studies in TCM syndromes using metabolomics and proteomics [4, 41]. In addition, in order to better serve the clinic, we tried to combine the model of epigenetic biomarkers and clinical symptoms, and finally, we achieved a better diagnostic model accuracy with 3 biomarkers (*SNORA59B*, *ZFAND3*, *APBA2*) plus 2 clinical symptoms (shortness of breath, night sweats). Our research indicates that the Epi-DS strategy based on 5hmC-Seal epigenetic sequencing can achieve precise diagnosis of QYDS in AF, providing an objective basis for TCM syndrome differentiation and typing studies, offering new insights for further research into molecular mechanisms, and making a significant contribution to the modernization and internationalization of TCM.

In summary, these results suggest that genome-wide 5hmC in plasma evDNAs can be considered as molecular markers for patients with AF, possessing the potential to differentiate between the QYDS and NQYDS phenotypes. In the diagnostic model, the 9 differential region-related genes are *SNORA59B*, *RCC1*, *GLIS1*, *FILIP1L*, *TFRC*, *PDLIM5*, *ZFAND3*, *CRYGN*, and *APBA2*. These genes are identified as key markers within the model, potentially playing a crucial role in distinguishing the characteristics of the syndrome and contributing to the diagnosis and treatment strategies in traditional Chinese medicine. *SNORA59B* is a subtype of small nucleolar RNA H/ACA box, and current research suggests that it may be involved in transcriptional regulation and is associated with immune dysfunction related to age-related diseases [92]. *RCC1*, as a highly conserved chromatin-associated protein and the only known guanine nucleotide exchange factor for Ran GTPase, plays a pivotal role in the regulation of the cell cycle [76, 93]. *GLIS1* serves as an activator or repressor of gene transcription and plays a crucial role in the regulation of various biological processes. It is involved in the reprogramming of aging stem cells and is associated with genomic stability [94, 95]. The overexpression of *FILIP1L* can lead to the inhibition of cell proliferation and migration, as well as an increase in apoptosis [78]. Additionally, one study has shown that it is associated with myocardial cell hypertrophy [96]. *TFRC*, also known as the transferrin receptor, is primarily involved in the transport of iron. Research indicates that it can mediate ferroptosis, which is involved in the treatment of cardiac injury, and it may also play a role in regulating macrophage infiltration and activation during heart failure [97, 98]. *PDLIM5* is a cytoskeleton-associated protein that plays a significant role in regulating cell proliferation, differentiation, and cell fate determination in a variety of tissues and cell types [80]. *ZFAND3*, acting as a transcription factor, is involved in a variety of cellular functions. It has been found to be associated with *GLIS3* in the context of type 2 diabetes [99]. Traditional Chinese medicine also posits a correlation between type 2 diabetes and QYDS [100]. *CRYGN* is primarily associated with the formation of the lens in the eye. Additionally, research has identified its involvement in the function of sensory neurons, suggesting a role in sensory perception and potentially in the maintenance of neural health and function [101]. *APBA2* is involved in the regulation of amyloid-β peptides, which are associated with a variety of neurological disorders, including Alzheimer’s disease, and depression [102, 103]. The functions of these DhMGs align with the functions of the QYDS-related modules annotated through WGCNA, which corroborates the reliability of our findings.

Overall, this study pioneers the use of 5hmC-Seal technology to explore traditional TCM syndromes from an epigenetic perspective and employs WGCNA and related network analyses for a comprehensive elucidation of the molecular functions of QYDS in the context of AF. Furthermore, it utilizes various machine learning algorithms to identify biomarkers for QYDS and constructs an accurate molecular diagnostic model, validated by an independent external cohort. Nevertheless, our study is not without limitations that warrant careful attention in future investigations. First, our Epi-DS pipeline centers on 5hmC-Seal as the core epigenetic assay, yet the regulatory landscape extends far beyond 5hmC alone. In addition to genome-wide 5mC profiling, a multidimensional view of the epigenome requires integrative data from histone modifications, chromatin accessibility, and three-dimensional architecture. Although 5hmC offers superior stability and detection sensitivity, deciphering the epigenetic mechanisms underlying AF in QYDS ultimately demands the seamless fusion of these diverse epigenomic technologies. Second, although we leveraged a comparatively large cohort for model training and validation, all samples were drawn from a single center, thereby limiting the generalizability that a multi-center design would afford. Moreover, we focused exclusively on one prevalent TCM pattern of AF. Future studies should not only expand the sample size but also encompass multiple AF-related TCM syndromes, and—critically—validate these findings across several independent centers. Additionally, although we have taken corresponding measures, the classification of patients into QYDS and NQYDS mainly depends on the physicians’ subjective judgment, which may lead to some bias in the results. Moreover, although DNA-based 5hmC is more stable than metabolites or proteins, the current 5hmC-Seal protocol remains costly. We therefore plan to develop a cost-effective diagnostic kit for AF-related QYDS using droplet digital PCR (ddPCR). Finally, our study has only provided a preliminary computational exploration of the molecular functions associated with AF-QYDS. The molecular functions identified in this study, such as the relative abnormalities in immune function and changes in energy metabolism, still require further experimental validation. Moreover, our study can only provide evidence that there is a certain correlation between TCM syndrome differentiation and epigenetics. The specific connections still need to be further confirmed through experiments. These aspects will be the focus of our future research to further elucidate the molecular mechanisms of QYDS.

## Conclusions

In summary, utilizing the 5hmC-Seal technology, we have constructed an epigenetic-level molecular network to elucidate the molecular functions of the TCM syndrome QYDS in AF and identified associated molecular biomarkers. Furthermore, we identified nine novel QYDS specific hydroxymethylation markers that were able to discriminate the QYDS from NQYDS. Additionally, we’ve built precise diagnostic models by integrating traditional Chinese medicine symptoms with modern molecular markers, offering a new direction for modern integrated traditional Chinese and Western medicine diagnosis. This study helps us to understand TCM syndromes from the perspective of modern biomedicine and contributes to the modernization and internationalization of TCM.

## Supplementary Information

Additional file 1.

Additional file 2.

Additional file 3.

## Data Availability

The data supporting the findings of this study are available from the corresponding author.

## Abbreviations

5hmC: 5‑Hydroxymethylcytosine
5mC: 5‑Methylcytosine
AF: Atrial Fibrillation
ANOVA: Analysis of Variance
APBA2: Amyloid Beta Precursor Protein Binding Family A Member 2
AUC: Area Under the Receiver Operating Characteristic Curve
BMI: Body Mass Index
cfDNA: Cell‑Free DNA
CHA₂DS₂‑VASc: Congestive heart failure, Hypertension, Age ≥75 (doubled), Diabetes, Stroke (doubled), Vascular disease, Age65–74, Sex category (female)
COPD: Chronic Obstructive Pulmonary Disease
CRYGN: Crystallin Gamma N
DBP: Diastolic Blood Pressure
ddPCR: Droplet Digital PCR
DhMG: Differentially Hydroxymethylated Gene
DhMR: Differentially Hydroxymethylated Region
Epi‑DS: Epigenetic Differential Syndrome
Epi‑DT: Epigenetic Diagnosis and Treatment
evDNA: Extracellular Vesicle DNA
EV: Extracellular Vesicle
FDR: False Discovery Rate
FILIP1L: Filamin A Interacting Protein 1‑Like
GLIS1: GLIS Family Zinc Finger 1
GO: Gene Ontology
GOBP: Gene Ontology Biological Process
GSEA: Gene Set Enrichment Analysis
HAS‑BLED: Hypertension, Abnormal renal/liver function, Stroke, Bleeding history or predisposition, Labile INR, Elderly (>65),Drugs/alcohol concomitantly
HDL‑C: High‑Density Lipoprotein Cholesterol
hMR: Hydroxymethylated Region
HR: Heart Rate
KEGG: Kyoto Encyclopedia of Genes and Genomes
LASSO: Least Absolute Shrinkage and Selection Operator
LDA: Linear Discriminant Analysis
LDL‑C: Low‑Density Lipoprotein Cholesterol
MACS: Model‑Based Analysis of ChIP‑Seq
MAPK: Mitogen‑Activated Protein Kinase
NQYDS: Non‑Qi‑Yin Deficiency Syndrome
PCA: Principal Component Analysis
PCI: Percutaneous Coronary Intervention
PDLIM5: PDZ and LIM Domain 5
PI3K: Phosphoinositide 3‑Kinase
QYDS: Qi‑Yin Deficiency Syndrome
RCC1: Regulator of Chromosome Condensation 1
RFECV: Recursive Feature Elimination with Cross‑Validation
ROC: Receiver Operating Characteristic
SBP: Systolic Blood Pressure
SGD: Stochastic Gradient Descent
SNORA59B: Small Nucleolar RNA, H/ACA Box 59B
SVM: Support Vector Machine
TCM: Traditional Chinese Medicine
TET2: Tet Methylcytosine Dioxygenase 2
TFRC: Transferrin Receptor
WGCNA: Weighted Gene Co‑expression Network Analysis
ZFAND3: Zinc Finger AN1‑Type Containing 3
