# Supplementary material for: Epigenetic precision diagnostics of traditional Chinese medicine (TCM) syndrome differentiation: a pilot study of atrial fibrillation with qi-yin deficiency syndrome based on 5-hydroxymethylcytosine signatures in extracellular vesicle DNA from plasma
Source: Chin Med. 2026 May 27;21:147. doi: 10.1186/s13020-025-01267-y (PMC13214416; doi:10.1186/s13020-025-01267-y)
Supplement: Supplementary file 1 — Additional file 1. [file 13020_2025_1267_MOESM1_ESM.docx]

**Supplementary material S1**

**Qi-Yin Deficiency Syndrome Scale**

Please answer the following questions based on feelings and symptoms over the past year.

| 1. Shortness of breath  A. Never B. Seldom appear (a little) C. Often appear D. Often E. Always |
| --- |
| 2. Lethargy in speech  A. Never B. Seldom appear (a little) C. Often appear D. Often E. Always |
| 3. Fatigue and lack of energy  A. Never B. Seldom appear (a little) C. Often appear D. Often E. Always |
| 4. Spontaneous or night sweats  A. Never B. Seldom appear (a little) C. Often appear D. Often E. Always |
| 5. Heat (palms, soles, chest)  A. Never B. Seldom appear (a little) C. Often appear D. Often E. Always |
| 6. Dizziness  A. Never B. Seldom appear (a little) C. Often appear D. Often E. Always |
| 7. Restlessness and irritability  A. Never B. Seldom appear (a little) C. Often appear D. Often E. Always |
| 8. Dry mouth  A. Never B. Seldom appear (a little) C. Often appear D. Often E. Always |
| 9. Dark red complexion on the cheeks  A. Never B. Seldom appear (a little) C. Often appear D. Often E. Always |
| 10. Insomnia and frequent dreams  A. Never B. Seldom appear (a little) C. Often appear D. Often E. Always |

At least two secondary symptoms should be considered, while also taking into account the tongue and pulse, integrating the four diagnostic methods for a comprehensive analysis.

**Supplementary material S2**

**The GSEA results pathways**


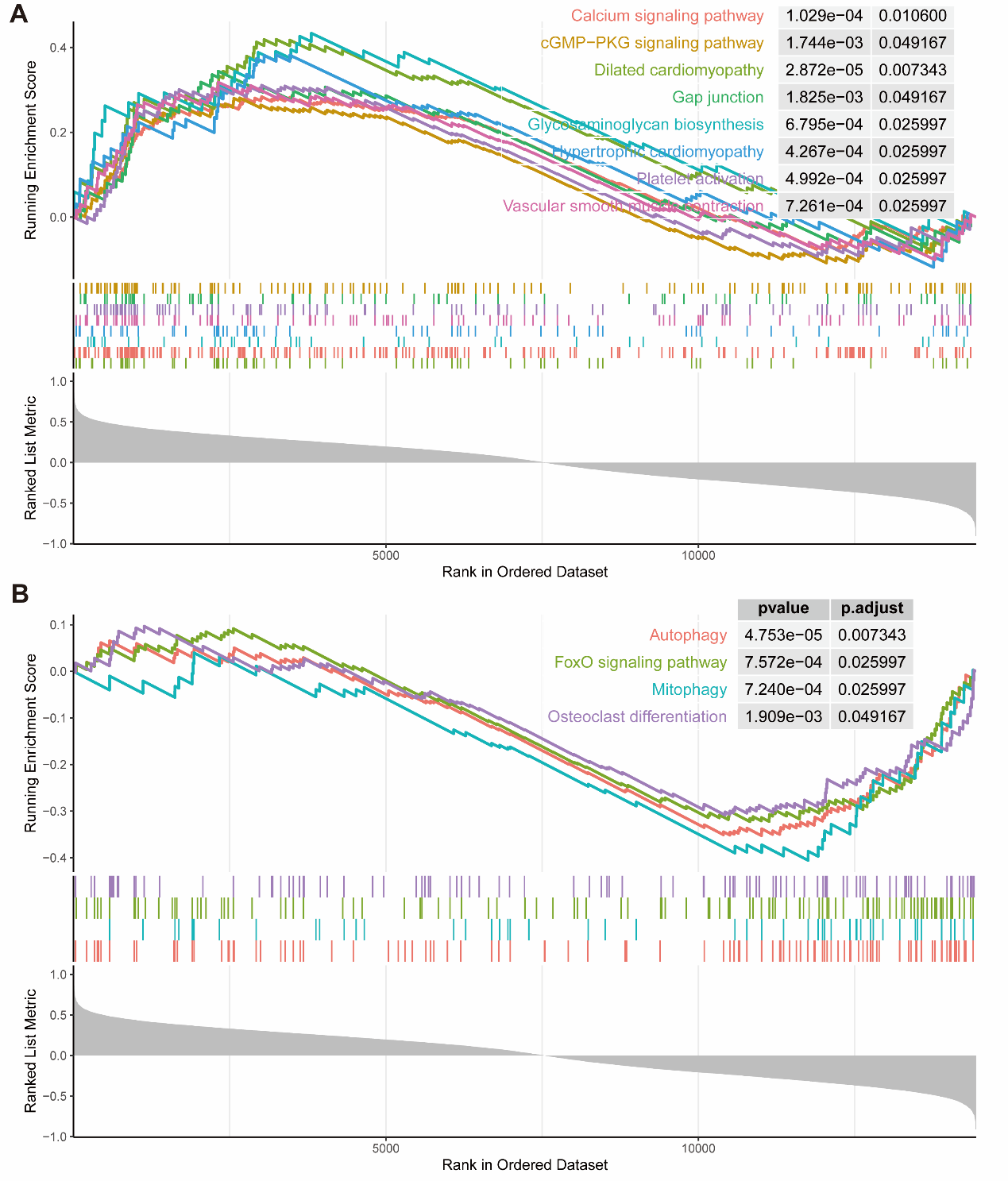


GSEAplot presents the GSEA curves for 12 distinct pathways. Figure A illustrates the eight upregulated pathways in QYDS, while Figure B delineates the four downregulated pathways within the same system.

**Supplementary material S3**

**Training Group 5hmC Differential Site Information Table**

This Excel spreadsheet contains relevant information about the differential sites in the training, including the starting position of the sequence, width, chromosome information, gene ID, transcript ID, pvalue, log_2_FC, distance to TSS (Transcription Start Site), ENSEMBL ID, gene symbol, and the full name of the gene.

**Supplementary material S4**

**Feature Selection and Diagnostic Model Construction**


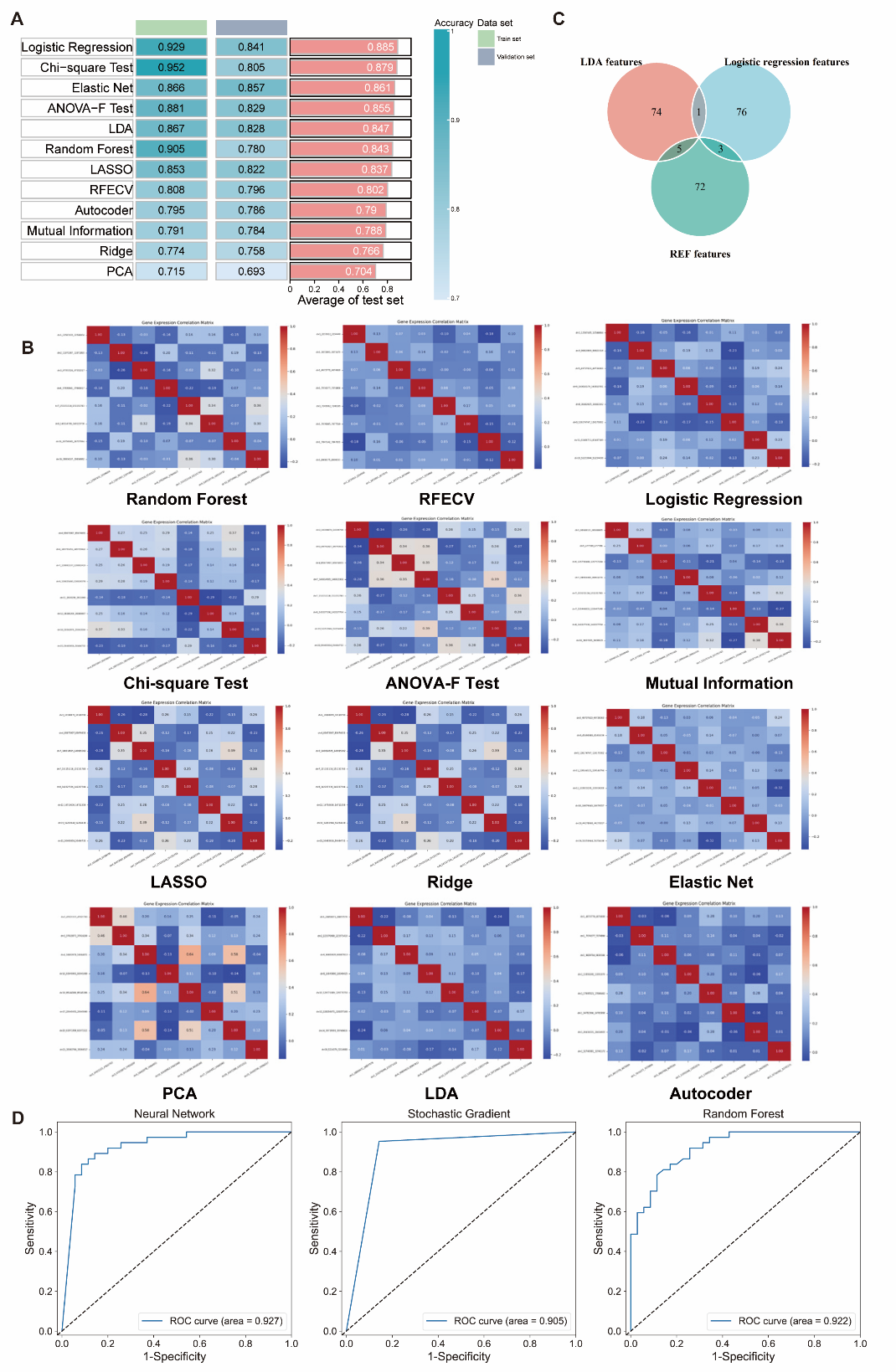


A shows the heat map of accuracy values for 12 machine learning algorithms in both the training and the validation. B displays the correlation heat map for the top 10 genes selected by the 12 algorithms. C illustrates the Venn diagram for the features ultimately selected. D presents the ROC curves for models independently built by the three algorithms on the external validation.

**Supplementary material S5**

**Diagnostic Model: Differential Region Information**

This Excel spreadsheet contains relevant information about the differential sites in the diagnostic model, including the starting position of the sequence, width, chromosome information, gene ID, transcript ID, pvalue, log_2_FC, distance to TSS (Transcription Start Site), ENSEMBL ID, gene symbol, and the full name of the gene.

**Supplementary material S6**

**Comparative Bar Chart of rehion-Related Genes in Diagnostic Models**


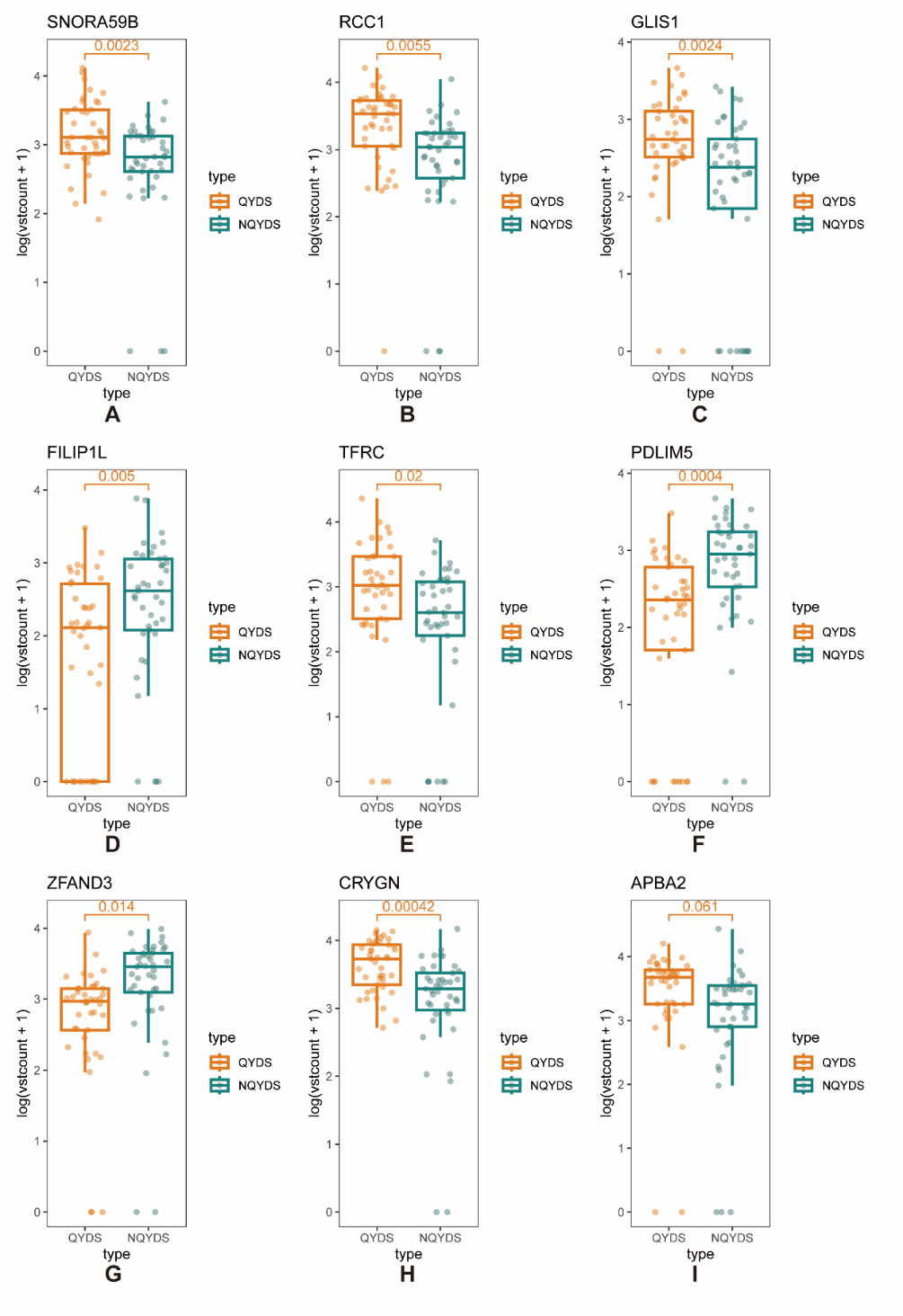


In the provided diagram, figures A to I depict the box plots for the differential region-associated genes within the training, with statistical testing performed using the t-test.

**Supplementary material S7**

**The individual ROC curves for each variant locus in the diagnostic model**
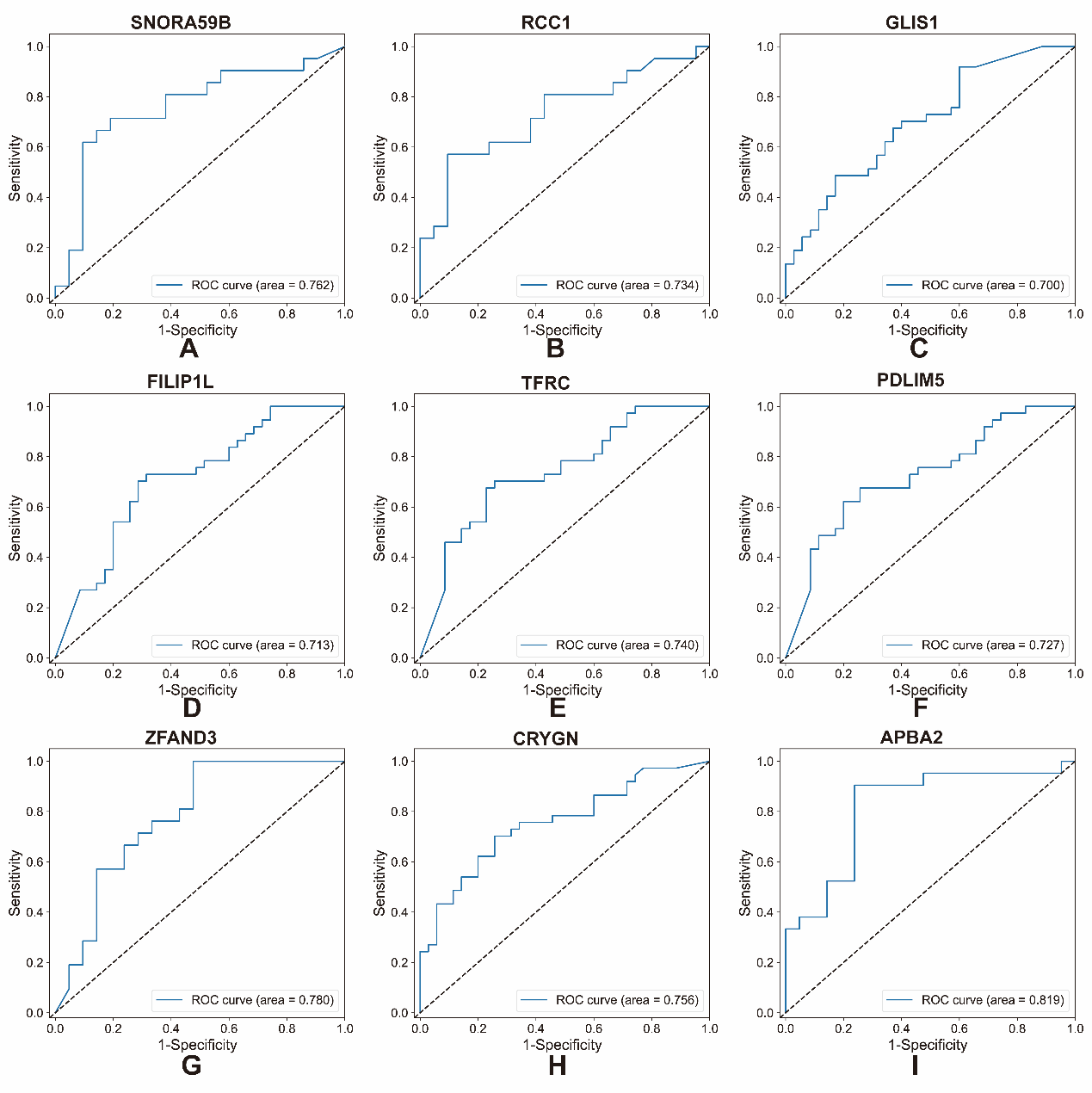


In the provided diagram, figures A to I represent the independent ROC curves for the differential region-associated genes within the diagnostic model. Each figure corresponds to a gene, illustrating its diagnostic performance through the ROC curve, which plots the true positive rate against the false positive rate at various threshold settings. The area under the curve (AUC) is a measure of the gene's ability to discriminate between disease states, with higher AUC values indicating better diagnostic accuracy.

**Supplementary material S8**

**Diagnosis Model-Related Information**


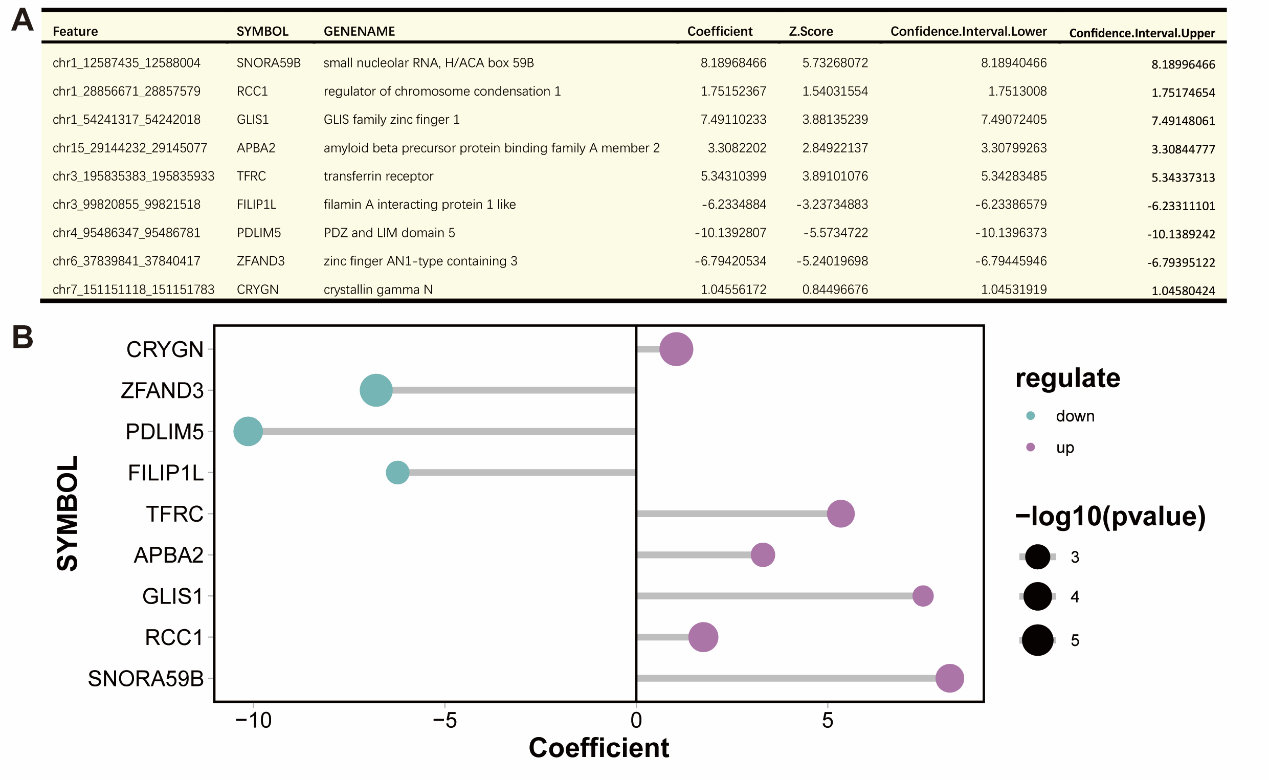


In the provided figure, Table A represents the relevant parameters within the diagnostic model, including information on the co-effect and other details. Figure B is a lollipop plot, where the horizontal axis represents the 'Coefficient', the vertical axis denotes the genes related to the difference regions, the size of the bubble corresponds to the P-value, and the color indicates the logFC (log Fold Change) value.

**Supplementary material S9**

**Sankey diagram of DhMGs In the Diagnostic Model**


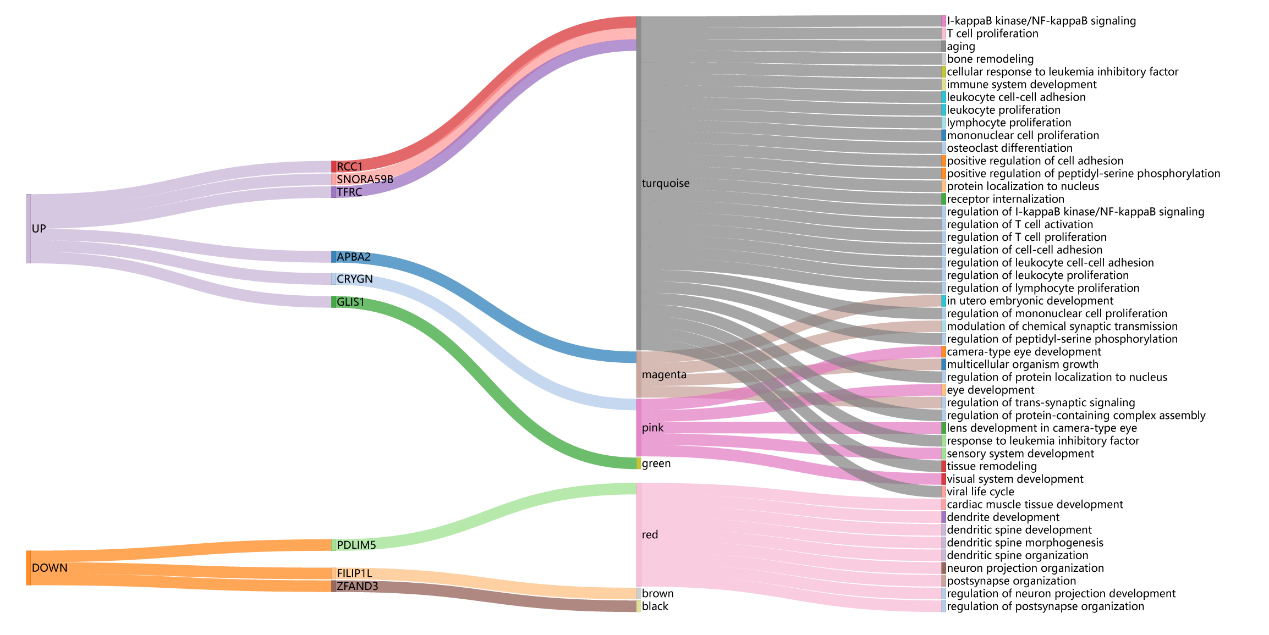


The diagram illustrates the expression patterns of the 9 DhMGs, their associated modules, and the GO (Gene Ontology) functions that these DhMGs are connected to.

**Supplementary material S10**

**Correlation analyses between the 9 DhMGs and clinical characteristics**


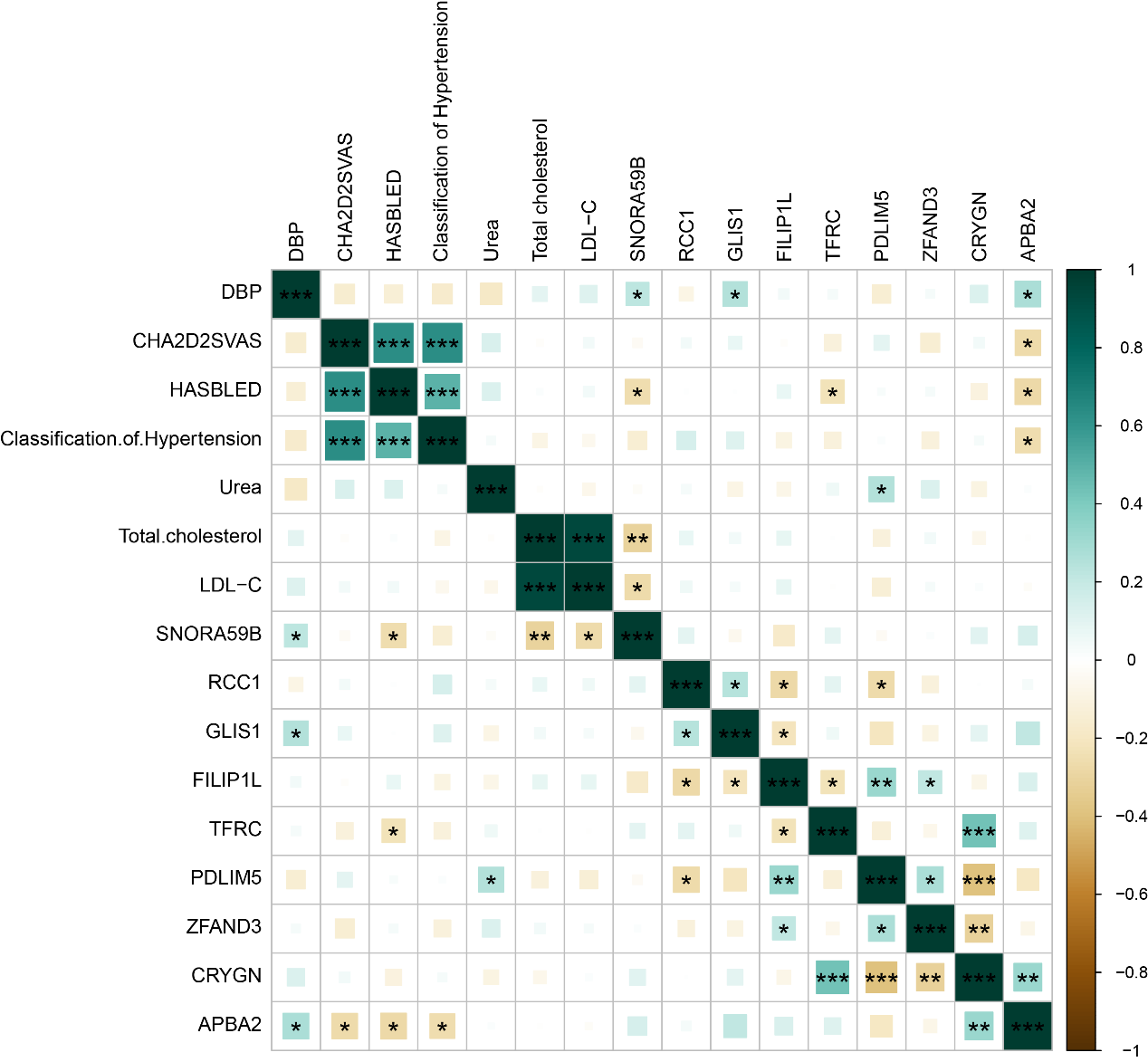


The diagram illustrates the correlation between the 9 DhMGs and clinical characteristics.
